# Streamlined Self-Collection Screening for Sexually Transmitted Infections and Human Papillomavirus: A Single-Group Secondary Analysis of a Randomized Clinical Trial

**DOI:** 10.1001/jamanetworkopen.2025.51345

**Published:** 2026-01-08

**Authors:** Anisha P. Ganguly, Peyton K. Pretsch, Noel T. Brewer, Lisa P. Spees, Michael G. Hudgens, Busola Sanusi, Lynn Barclay, Alicia Carter, Stephanie B. Wheeler, Jennifer S. Smith

**Affiliations:** 1 Department of Internal Medicine, School of Medicine, University of North Carolina at Chapel Hill; 2 Lineberger Comprehensive Cancer Center, University of North Carolina at Chapel Hill; 3 Department of Epidemiology, Gillings School of Global Public Health, University of North Carolina at Chapel Hill; 4 Department of Health Behavior, Gillings School of Global Public Health, University of North Carolina at Chapel Hill; 5 Division of Pharmaceutical Outcomes and Policy, Eshelman School of Pharmacy, University of North Carolina at Chapel Hill; 6 Department of Biostatistics, Gillings School of Global Public Health, University of North Carolina at Chapel Hill; 7 American Sexual Health Association, Durham, North Carolina; 8 Laboratory Corporation of America Holdings, Burlington, North Carolina; 9 Department of Health Policy and Management, Gillings School of Global Public Health, University of North Carolina at Chapel Hill

## Abstract

**Importance:**

Human papillomavirus (HPV) self-collection increases cervical cancer screening uptake among women underscreened for cervical cancer, particularly those from marginalized low-income and racial and ethnic backgrounds. Underscreened women are also at high risk for other sexually transmitted infections (STIs) that can be similarly screened via self-collection.

**Objective:**

To evaluate an intervention streamlining testing for other STIs alongside HPV self-collected samples among low-income women.

**Design, Setting, and Participants:**

This is a secondary analysis of the My Body, My Test–3 study, a randomized clinical trial testing a mailed self-collection intervention to improve cervical cancer screening. The My Body, My Test–3 study was conducted from April 2016 to December 2019 in 22 counties in North Carolina among low-income women overdue for cervical cancer screening. This analysis included participants randomized to the trial intervention group with valid STI and HPV results. Data analysis occurred from October 2024 to February 2025.

**Intervention:**

The intervention included a mailed self-collection kit and instructions to self-collect a cervicovaginal sample. Samples were tested for other STIs and HPV using the Aptima assay.

**Main Outcomes and Measures:**

The primary outcome was a positive test result for other STIs (including chlamydia, gonorrhea, and trichomoniasis). A risk factor analysis was conducted to identify factors associated with testing positive for other STIs. Secondary outcomes included rate of follow-up care and perceptions of self-collection among participants with positive STI results.

**Results:**

Among 327 participants (median [IQR] age, 42 [25-63] years; 38 [8.6%] Hispanic, 146 [44.7%] non-Hispanic Black, and 133 [40.7%] non-Hispanic White), 51 (15.6%) tested positive for other STIs and 51 (15.6%) tested positive for HPV; 7 (2.1%) tested positive for both. Risk factors for other STIs included non-Hispanic Black race and ethnicity compared with non-Hispanic White race and ethnicity (adjusted odds ratio [aOR], 4.1; 95% CI, 1.5-11.6), having 2 or more sexual partners in the last year compared with having none (aOR, 5.7; 95% CI, 1.0-31.4), single marital status compared with married or partnered status (aOR, 5.6; 95% CI, 1.1-27.9), and current smoking compared with none (aOR, 4.1; 95% CI, 1.7-10.4). Among participants who tested positive for other STIs, 34 (66.7%) received follow-up care. Most participants (130 [84.4%]) preferred testing for both HPV and other STIs in the future.

**Conclusions and Relevance:**

In this secondary analysis of a randomized clinical trial of 327 participants, nearly 1 in 6 tested positive for other STIs via streamlined testing in a mailed HPV self-collection intervention. Self-collection may improve both cervical cancer and STI screening for women from marginalized backgrounds.

**Trial Registration:**

ClinicalTrials.gov Identifier: NCT02651883

## Key Points

**Question:**

What is the prevalence of human papillomavirus (HPV) and other sexually transmitted infections (STIs) with simultaneous testing of mailed self-collection kits in a population of low-income women?

**Findings:**

In this secondary analysis of a randomized clinical trial including 327 intervention participants, nearly 1 in 6 participants tested positive for other STIs, the same rate as those positive for HPV.

**Meaning:**

These findings suggest that streamlining testing for both HPV and other STIs may address multiple preventive care needs among underscreened women.

## Teaser

This secondary analysis of a randomized clinical trial evaluates whether self-testing for chlamydia, gonorrhea, and trichomoniasis at the same time as human papillomavirus improves uptake of testing among underscreened women.

## Introduction

Cervical cancer remains an important public health problem in the United States and globally, largely due to inadequate access to screening. Risk factors for nonadherence to cervical cancer screening in the United States include lack of health insurance, cost, logistical barriers to attending clinical appointments, minoritized racial and ethnic background, and health-related social needs resulting from structural inequities.^1,2,3,4,5^ In addition to high-risk types of human papillomavirus (HPV), which cause cervical cancer, other sexually transmitted infections (STIs) remain prevalent in the United States and other countries. Most recent estimates from the United States indicate 600 000 cases of gonorrhea, 1.6 million cases of chlamydia, and more than 1 million cases of trichomoniasis yearly, with rates increasing over the past decade.^6,7^ Of note, while chlamydia and gonorrhea infections are reportable STIs,^8^ no states currently require reporting of trichomoniasis, which may underestimate its true prevalence.^9^ Risk factors for HPV infection^1,10,11,12^ are similar for other STIs.^13,14,15^

Self-collection testing for human papillomavirus (HPV) has emerged as a significant innovation to improve access to cervical cancer screening.^16,17,18^ Individuals use a brush or swab to obtain their own cervicovaginal sample to be tested for high-risk HPV.^18^ Self-collection improves adherence to recommended cervical cancer screening and increases the early detection of cervical dysplasia among women overdue for screening.^18,19,20^ Mailed HPV self-collection is a particularly effective strategy to reach women overdue for cervical cancer screening to improve screening uptake, particularly for those with limited health care access or barriers to clinic-based screening.^16,21,22,23^

The United States Preventive Services Task Force (USPSTF), the American College of Obstetrics and Gynecology (ACOG), and the American Society for Colposcopy and Cervical Pathology (ASCCP) recommend regular cervical cancer screening for women ages 21 to 65 years through cervical cytology every 3 years. Alternatively, women ages 30 to 65 years old may receive HPV testing every 5 years or combination HPV testing and cytology (cotesting).^24,25,26^ Regarding STI screening, the USPSTF recommends screening for chlamydia and gonorrhea among sexually active women age 24 years or younger and women age 25 years or older at higher risk for STIs.^27^ Previous research has shown that self-collection for STIs other than HPV increases uptake of STI screening.^28,29^ Individual meta-analyses have shown that HPV self-collection doubles cervical cancer screening uptake^18^ and that STI self-collection increases guideline-recommended STI screening by nearly 3-fold^28^ compared with usual clinic-based care. However, studies in the United States have yet to evaluate the potential of self-collection to streamline screening for both HPV and other STIs. Exploration of self-collection for both HPV and other STIs is needed to understand how streamlined testing may address multiple preventive care gaps at once and improve sexual health among women historically underscreened for cervical cancer and STIs.

Our study objective was to evaluate STI screening alongside mailed HPV self-collection among low-income women overdue for cervical cancer screening. We aimed to report STI results, understand risk factors for testing positive for STIs other than HPV, and measure follow-up outcomes after positive test results among participants testing positive for other STIs to inform future self-collection efforts for STI screening in similar high-risk populations.

## Methods

### Study Sample

We conducted a single-group secondary analysis of the My Body, My Test–3, a phase 3, open-label, 2-group randomized clinical trial conducted in 22 counties in North Carolina from 2016 to 2019. The trial consisted of a mailed HPV self-collection intervention to increase uptake of cervical cancer screening (Supplement 1).^30,31,32^ Participants were eligible for inclusion if they were aged 25 to 64 years; had an intact cervix; were uninsured or insured by Medicaid or Medicare; reported an income of 250% or less of the US Federal Poverty Level; and resided in the catchment area of 21 collaborating clinics in North Carolina for follow-up care. Additionally, participants were eligible only if they were overdue for cervical cancer screening, defined as 6 or more years since their last physician-collected HPV test or cotest or 4 or more years since their last cervical cytology test.

Written consent was reviewed and obtained from eligible women, and Heath Information Portability and Accountability Act forms were sent via mail. Before study enrollment, all required forms had to be completed and returned. This study followed the Consolidated Standards of Reporting Trials (CONSORT) reporting guideline for randomized clinical trials^33^ and was reviewed and approved by the University of North Carolina institutional review board.

All enrolled participants were randomly assigned in a 2:1 ratio to receive a mailed self-collection kit and assistance in scheduling appointments for clinic-based cervical cancer screening (intervention group) or to receive scheduling assistance alone (control group).^30^ Separate randomization lists were generated per county, using permuted blocks of 9 patients (6:3 intervention to control) to ensure that the proportion of individuals assigned to the intervention was similar across counties. Participating women and trial staff were not blinded to randomization assignment. A priori sample size calculation determined that 510 participants in a 2:1 randomization ratio of intervention to control would provide 88% to 94% power to detect a 15% or greater difference between groups for the primary outcome of cervical cancer screening uptake, assuming a rate of screening uptake of 60% to 80% in the intervention group. Ultimately 697 patients were enrolled and randomized.^31^ In this single-group secondary analysis, we restricted the analytic sample to participants in the intervention group (n = 461) who returned a self-collection kit (n = 341) and had valid STI and HPV results (n = 327) (eFigure 1 in Supplement 2).^31^

### Study Procedures

Participants in the intervention group were mailed an HPV self-collection kit containing a Viba-Brush (Rovers Medical Devices) and a vial containing 4.3 mL of Aptima sample transport media (Hologic), approved for sample stability for testing up to 60 days at room temperature and transport via mail. Patients received patient education in English or Spanish about HPV testing, cervical cancer screening, and STI testing as well as illustrated instructions for self-collection and mail return (eMethods in Supplement 2). Self-collection kits were mailed back and sent to Labcorp in Burlington, North Carolina, for STI testing.^32^ Samples were tested for *Chlamydia trachomatis*, *Neisseria gonorrheae*, and *Trichomonas vaginalis *using the Hologic Aptima assay nucleic acid amplification tests. Samples were tested for *C trachomatis* and *N gonorrheae* based on USPSTF screening guidelines.^27,34^ Although there is currently insufficient evidence for USPSTF-recommended screening for trichomoniasis, *T vaginalis* was included in testing due to prior research suggesting a positive association between trichomoniasis and HPV-associated cervical dysplasia.^35,36^ Participants who did not return a self-collection kit within 3 weeks received a reminder letter and after an additional 2 weeks received a reminder telephone call. Participants received their STI results by telephone call.

Study staff offered participants assistance in scheduling appointments for clinic-based cervical cancer screening at a study-affiliated community clinic, irrespective of trial group or self-collection results, where participants could receive cervical cancer screening as well as follow-up care for positive STI results. Staff made up to 3 telephone call attempts to reach participants to provide scheduling assistance for a clinic-based cervical cancer screening appointment. Participants who tested positive for other STIs were also referred to the health department for treatment.

Participants completed a telephone-administered questionnaire at baseline to collect sociodemographic, behavioral, and clinical data. Participants also received a follow-up questionnaire 1 week after delivery of laboratory results from self-collection kits to assess knowledge and attitudes about cervical cancer screening and self-collection. The follow-up questionnaire also assessed receipt of follow-up care after positive STI result. Questionnaires were administered by telephone to ensure completeness of data collection among trial participants recruited from a wide catchment area and to mitigate health literacy barriers to complete the questionnaire. Questionnaire items utilized simple, patient-centered language developed in phases 1 and 2 My Body, My Test studies.^21,37^ Participants received $25 in compensation for completing each questionnaire regardless of clinical participation or screening completion.

### Statistical Analysis

We calculated the prevalence of HPV and other STIs (*C trachomatis*, *N gonorrhea*, and *T vaginalis*) and reported the distribution of positive laboratory results obtained through self-collection. Risk factors for testing positive for other STIs were then examined with logistic regression to calculate age-adjusted odds ratios (ORs) and corresponding 95% CIs to compare baseline characteristics among participants who tested positive and negative for other STIs. A subsequent multivariable model adjusted for multiple risk factors associated with other STIs in the age-adjusted model: age, race and ethnicity, number of sexual partners in the last year, marital status, current smoking, overuse of alcohol and drugs, age of first vaginal intercourse, self-rated mental health, receipt of social assistance, and time to travel to appointments. Race and ethnicity were self-reported. Participants selected from yes, no, or unsure for Latina/Hispanic ethnicity and from American Indian or Alaska Native, Asian, Black or African American, Native Hawaiian or Pacific Islander, and White for race. Due to small sample sizes, American Indian or Alaska Native, Asian, and Native Hawaiian or Pacific Islander individuals as well as those indicating an additional non-Hispanic identity were included in the additional racial groups category. Information on race and ethnicity was collected to understand the health equity implications of STI self-collection as an intervention to improve screening for HPV and other STIs. Lack of multicollinearity among risk factors was confirmed by variance inflation factors less than 5. All statistical tests were 2-sided, and statistical significance was defined at α < .05.

We then measured rates of follow-up care after positive STI test within 6 months of trial enrollment. Follow-up care was defined as self-reported receipt of other STI treatment on follow-up questionnaire and/or attendance at the clinic-based appointment for cervical cancer screening offered with scheduling assistance through the study. Follow-up care after a positive STI test was stratified by race and ethnicity. We also assessed perceptions of self-collection among participants testing positive for other STI, HPV, or no STI. Patients who tested negative for all STIs were included in perceptions outcomes due to prior research that has suggested that individuals with negative test results rate self-collection most favorably.^38,39^ Data analysis occurred October 2024 to February 2025 using SAS version 9.4 (SAS Institute).

## Results

Among 327 participants who returned a self-collection kit and had valid STI results, the median (IQR) age was 42 (25-63) years. Overall, 28 participants (8.6%) identified as Hispanic, 146 (44.7%) as non-Hispanic Black, 133 (40.7%) as non-Hispanic White, and 20 (6.1%) as belonging to an additional racial group. Most participants (258 [78.9%]) were uninsured, 62 (18.9%) received Medicaid, and 7 (2.1%) received Medicare or were dual-eligible. Fifty-one participants (15.6%) tested positive for HPV, and 51 (15.6%) tested positive for other STIs, of whom 7 (2.1%) were positive for both HPV and other STIs (Table 1; eFigure 2 in Supplement 2). Among the 51 participants who tested positive for other STIs, 45 (88.2%) tested positive for *T vaginalis*, 7 (13.7%) tested positive for *C trachomatis*, and 1 (2.0%) tested positive for *N gonorrhea*. Nine participants tested positive for multiple STIs: 6 (66.7%) for both HPV and *T vaginalis*, 2 (22.2%) for both *T vaginalis* and *C trachomatis*, and 1 (11.1%) for both HPV and *N gonorrhea*.

**Table 1.** Baseline Characteristics of Participants, Stratified by Sexually Transmitted Infections Other Than HPV

Characteristic	Participants, No. (%)		
	Overall (N = 327)	Other STI^a^	
		Yes (n = 51)	No (n = 276)
Age, median (IQR), y	42 (25-63)	40 (26-58)	43 (25-63)
High-risk HPV RNA positivity	51 (15.6)	7 (13.7)	44 (15.9)
Trichomoniasis positivity	45 (13.8)	45 (88.2)	0
*Chlamydia trachomatis* positivity	7 (2.1)	7 (13.7)	0
Gonorrhea positivity	1 (0.3)	1 (2.0)	0
Race and ethnicity			
Hispanic	28 (8.6)	3 (5.9)	25 (9.1)
Non-Hispanic Black	146 (44.7)	35 (68.6)	111 (40.2)
Non-Hispanic white	133 (40.7)	8 (15.7)	125 (45.3)
Additional groups^b^	20 (6.1)	5 (9.8)	15 (5.4)
Insurance status			
Uninsured	258 (78.9)	36 (70.6)	222 (80.4)
Medicaid	62 (18.9)	15 (29.4)	47 (17.0)
Medicare and/or dual eligible	7 (2.1)	0	7 (2.4)
Income, median (IQR), $	15 000 (8400-25 000)	14 400 (2400-20 000)	16 400 (8400-25 000)
Sexual orientation^c^			
Heterosexual or straight	297 (91.7)	43 (84.3)	254 (93.0)
Gay or lesbian	9 (2.8)	0	9 (3.3)
Bisexual	18 (5.6)	8 (15.7)	10 (6.7)
No. of sexual partners in last year^c^			
0	56 (17.3)	4 (8)	52 (19.1)
1	193 (59.8)	19 (38.0)	174 (63.7)
≥2	72 (22.3)	27 (54.0)	45 (16.5)
Never had sex	2 (0.6)	0	2 (0.7)
Marital status^c^			
Single or never married	151 (46.6)	35 (68.6)	116 (42.5)
Married or living with partner	81 (25.0)	3 (5.9)	78 (28.6)
Divorced, separated, or widowed	92 (28.4)	13 (25.5)	79 (28.9)
Current smoker^c^			
No	189 (58.5)	14 (17.5)	175 (64.3)
Yes	134 (41.5)	37 (72.6)	97 (35.7)
No. of health care professional visits in the past year, median (IQR)	1 (0-2)	1 (0-2)	1 (0-2)
Frequency of condom use			
Never	142 (43.4)	14 (27.5)	128 (46.4)
Rarely or sometimes	56 (17.1)	13 (25.5)	43 (15.6)
Half or most of the time	33 (10.1)	8 (16.7)	25 (9.1)
Always	55 (16.8)	12 (23.5)	43 (15.6)
Not sexually active or not currently having sex	41 (12.5)	4 (7.8)	37 (13.4)
Self-reported overuse of alcohol or drugs^d^	60 (18.4)	16 (31.4)	44 (15.9)
Age of first vaginal intercourse, y^c^			
<16	125 (38.5)	31 (60.8)	94 (34.3)
≥16	198 (60.9)	20 (39.2)	178 (65.0)
Never had sex	2 (0.6)	0	2 (0.7)
Self-rated physical health			
Excellent	19 (5.8)	1 (2.0)	18 (6.5)
Very good	87 (26.6)	11 (21.6)	76 (27.5)
Good	106 (32.4)	16 (31.4)	90 (32.6)
Fair	100 (30.6)	21 (41.2)	79 (28.6)
Poor	15 (4.6)	2 (3.9)	13 (4.7)
Self-rated mental health^c^			
Excellent	68 (20.9)	9 (17.7)	59 (21.5)
Very good	101 (31.0)	10 (19.6)	91 (33.1)
Good	73 (22.7)	12 (23.5)	61 (22.2)
Fair	74 (22.7)	17 (33.3)	57 (20.7)
Poor	10 (3.1)	3 (5.9)	7 (2.6)
Receipt of social assistance (%)^e^	160 (49.7)	36 (72.0)	124 (45.6)
Mode of transportation for appointments^c^			
Public transportation	102 (31.3)	18 (35.3)	84 (30.4)
Own car	186 (57.1)	25 (49.0)	161 (58.6)
Get a ride from someone for free	23 (7.1)	5 (9.8)	18 (6.6)
Pay someone you know to get a ride	10 (3.1)	2 (3.9)	8 (2.9)
Taxi or ride share	2 (0.6)	1 (2.0)	1 (0.4)
Walk	3 (09)	0	3(1.1)
Time to travel to appointment, min^c^			
0-15	60 (20.8)	6 (13.6)	54 (22.1)
16-30	137 (47.6)	20 (45.5)	117 (48.0)
31-60	58 (20.4)	9 (20.5)	49 (20.1)
≥61	33 (11.5)	9 (20.5)	24 (9.8)

Abbreviations: HPV, human papillomavirus; STI, sexually transmitted infection.

^a^ Other STIs included *Chlamydia trachomatis*, *Neisseria gonorrhea*, and *Trichomonas vaginalis* infections.

^b^ Additional racial and ethnic groups included 2 American Indian or Alaska Native individuals, 3 Asian individuals, 2 Native Hawaiian or Pacific Islander individuals, and 13 individuals with additional non-Hispanic identities.

^c^ Missing responses from baseline questionnaire: sexual orientation (n = 3), number of sexual partners in last year (n = 4), marital status (n = 3), current smoker (n = 4), age of first vaginal intercourse (n = 2), self-rated mental health (n = 1), mode of transportation for appointments (n = 1), time to travel to appointment (n = 39).

^d^ Self-reported overuse of alcohol or drugs was defined by answering yes to the question, “In the last year, have you ever drunk alcohol or used drugs more than you meant to?”

^e^ Social assistance programs included food stamps, housing assistance, social security, supplemental security income, disability payments, or other public benefits.

Age-adjusted risk factors for positivity to other STIs included non-Hispanic Black race and ethnicity (age-adjusted OR [aOR], 5.6 [95% CI, 2.5-12.8]) and additional racial groups (aOR, 4.8 [95% CI, 1.4-16.9]) compared with non-Hispanic White race and ethnicity; 2 or more sexual partners reported in the last year (aOR, 6.3 [95% CI, 2.0-20.1]) compared with 0 partners; single marital status (aOR, 7.9 [95% CI, 2.3-26.9]) or being divorced, separated, or widowed (aOR, 5.0 [95% CI, 1.3-18.6]) compared with married or partnered marital status; current smoking (aOR, 4.7 [95% CI, 2.4-9.2]) compared with no smoking; self-reported overuse of alcohol or drugs (aOR, 2.4 [95% CI, 1.2-4.7]) compared with none; age of first vaginal intercourse younger than 16 years (aOR, 2.8 [95% CI, 1.5-5.3]) compared with age 16 years or older; fair or poor self-rated mental health (aOR, 2.1 [95% CI, 1.1-4.0]) compared with excellent, very good, or good self-rated mental health; receipt of social assistance (aOR, 3.4 [95% CI, 1.7-6.6]) compared with none; and more than 1 hour of travel time for appointments (aOR, 3.2 [95% CI, 1.0-10.1]) compared with 15 minutes or less (Table 2). In the multivariable model, non-Hispanic Black race and ethnicity (multivariable OR [mOR], 4.1 [95% CI, 1.5-11.6]) compared with non-Hispanic White race and ethnicity; 2 or more sexual partners (mOR, 5.7 [95% CI, 1.0-31.4]) compared with none; single marital status (mOR, 5.6 [95% CI, 1.1-27.9]) compared with married or partnered marital status; and current smoking (mOR, 4.1 [95% CI, 1.7-10.4]) compared with none remained significant risk factors for positivity to other STIs.

**Table 2.** Age-Adjusted and Multivariable Adjusted Risk Factor Analysis for Positive Test for Other STIs

Risk factor	No. with other STI/total No. (%)^a^	OR (95% CI)	
		Age-adjusted	Multivariable adjusted^b^
Overall	51/327 (15.6)	NA	NA
Age, y			
25-34	16/89 (18.0)	1.7 (0.7-3.9)	1.8 (0.6-6.0)
35-49	24/141 (17.0)	1.6 (0.7-3.5)	1.5 (0.5-4.8)
50-65	11/97 (21.6)	1 [Reference]	1 [Reference]
Race and ethnicity			
Hispanic	3/28 (10.7)	1.9 (0.5-7.6)	2.7 (0.4-18.2)
Non-Hispanic Black	35/146 (24.0)	5.6 (2.5-12.8)^c^	4.1 (1.5-11.6)^c^
Non-Hispanic White	8/133 (6.0)	1 [Reference]	1 [Reference]
Additional groups^d^	5/20 (25.0)	4.8 (1.4-16.9)^c^	1.2 (0.2-5.8)
Insurance status			
Uninsured	36/258 (14.0)	1 [Reference]	NA
Medicaid	15/62 (24.2)	1.9 (0.9-3.8)	NA
Medicare and/or dual eligible^e^	0/7	NA	NA
Income, $			
<$10 000	17/100 (17.0)	1.6 (0.7-3.6)	NA
$10 000-$25 000	22/126 (17.5)	1.7 (0.7-4.0)	NA
>$25 000	10/85 (11.8)	1 [Reference]	NA
Sexual orientation			
Heterosexual or straight	43/297 (14.5)	1 [Reference]	NA
Gay, lesbian, bisexual, or other	8/27 (29.6)	2.1 (0.9-5.3)	NA
No. of sexual partners in last year			
0	4/56 (7.1)	1 [Reference]	1 [Reference]
1	19/193 (9.8)	1.2 (0.4-3.8)	2.0 (0.4-10.8)
≥2	27/72 (37.5)	6.3 (2.0-20.1)^c^	5.7 (1.0-31.4)^c^
Never had sex	0/2	NA	NA
Marital status			
Single or never married	35/151 (23.2)	7.9 (2.3-26.9)^c^	5.6 (1.1-27.9)^c^
Married or living with partner	3/81 (3.7)	1 [Reference]	1 [Reference]
Divorced, separated, or widowed	13/92 (14.1)	5.0 (1.3-18.6)^c^	4.1 (0.7-23.5)
Current smoker			
No	14/189 (7.4)	1 [Reference]	1 [Reference]
Yes	37/134 (27.6)	4.7 (2.4-9.2)^c^	4.1 (1.7-10.4)^c^
Frequency of condom use^f^			
Never	14/142 (9.9)	0.4 (0.1-0.9)	NA
Rarely or sometimes	13/56 (23.2)	1.1 (0.4-2.7)	NA
Half or most of the time	8/33 (24.2)	1.1 (0.4-3.0)	NA
Always	12/55 (21.8)	1 [Reference]	NA
Not currently sexually active	4/41 (9.8)	NA	NA
Self-reported overuse of alcohol or drugs^g^			
No	35/267 (13.1)	1 [Reference]	1 [Reference]
Yes	16/60 (26.7)	2.4 (1.2.-4.7)^c^	1.3 (0.5-3.5)
Age of first vaginal intercourse, years			
<16	31/125 (24.8)	2.8 (1.5-5.3)^c^	1.5 (0.6-3.8)
≥16	20/198 (10.1)	1 [Reference]	NA
Never had sex	0/2	NA	NA
Self-rated physical health			
Excellent, very good, or good	28/212 (13.2)	1 [Reference]	NA
Fair or poor	23/115 (20.0)	1.7 (0.9-3.2)	NA
Self-rated mental health			
Excellent, very good, or good	31/242 (12.8)	1 [Reference]	1 [Reference]
Fair or poor	20/84 (23.8)	2.1 (1.1-4.0)^c^	1.5 (0.6-3.6)
Receipt of social assistance^h^			
No	14/162 (8.6)	1 [Reference]	1 [Reference]
Yes	36/160 (22.5)	3.4 (1.7-6.6)^c^	2.3 (0.9-5.7)
Time to travel to appointment, min			
0-15	6/60 (10.0)	1 [Reference]	1 [Reference]
16-30	20/137 (14.6)	1.4 (0.5-3.7)	1.0 (0.3-3.6)
31-60	9/58 (15.5)	1.5 (0.5-4.6)	1.0 (0.2-4.1)
≥60	9/33 (27.3)	3.2 (1.0-10.1)^c^	2.1 (0.5-9.6)

Abbreviations: NA, not applicable; STI, sexually transmitted infections.

^a^ Other sexually transmitted infections included *Chlamydia trachomatis*, *Neisseria gonorrhea*, and *Trichomonas vaginalis* infections.

^b^ Multivariable analysis adjusted for all statistically significant variables from age-adjusted analysis.

^c^ Statistically significant at α < .05.

^d^ Additional racial and ethnic groups included 2 American Indian or Alaska Native individuals, 3 Asian individuals, 2 Native Hawaiian or Pacific Islander individuals, and 13 individuals with other non-Hispanic identities.

^e^ Dual eligible individuals received Medicare with Medicaid supplemental insurance.

^f^ Although associated with positive test for other STI in the age-adjusted model, frequency of condom use was not included in the multivariable model due to collinearity with marital status.

^g^ Self-reported overuse of alcohol or drugs was defined by answering yes to the question, “In the last year, have you ever drunk alcohol or used drugs more than you meant to?”

^h^ Social assistance was defined as supplemental nutritional assistance program (colloquially known as food stamps), housing assistance, social security, supplemental security income, disability payments, or other public benefits.

Among 51 participants positive for other STIs, including 7 participants also positive for HPV, delivery of laboratory results and scheduling assistance for a clinic-based cervical cancer screening appointment was 98% (50 participants) (Figure). Thirty-four participants (66.7%) who tested positive for other STIs received follow-up care, of whom 31 (91.2%) self-reported receiving STI treatment and 12 (35.3%) attended the clinic-based appointment offered through the study. Because race and ethnicity emerged as risk factors for positivity to other STIs in the multivariable risk factor analysis, we stratified rates of follow-up care after positive test results among non-Hispanic Black participants, non-Hispanic White participants, and participants belonging to additional racial groups to evaluate equitable follow-up of positive STI results. Among 51 participants positive for other STI, 35 (68.6%) identified as non-Hispanic Black, 8 (15.7%) identified as non-Hispanic White, and 8 (15.7%) belonged to additional groups. The rate of follow-up care among participants testing positive for other STIs was 24 of 35 (68.6%) among non-Hispanic Black participants, 4 of 8 (50.0%) among non-Hispanic White participants, and 6 of 8 (75.0%) among participants who belonged to additional racial groups.

**Figure.**
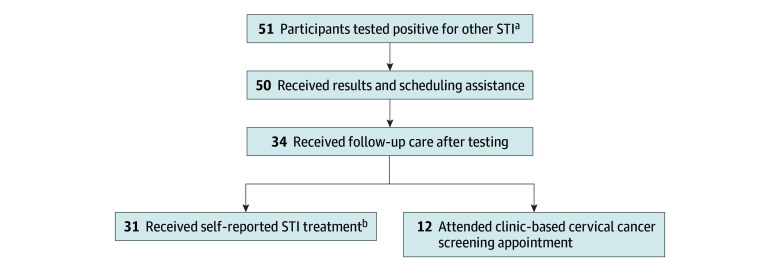
Follow-up Care Among Participants After Positive Test for Sexually Transmitted Infections (STI) Among 51 participants positive for other sexually transmitted infections, 50 (98%) received results from positive STI test and scheduling assistance for a clinic-based appointment, and 34 (67%) received follow-up care after positive STI test. ^a^A total of 7 participants tested positive for both other STIs and human papillomavirus. ^b^A total of 9 participants received both STI treatment and clinic-based cervical cancer screening.

Perceptions of self-collection were explored among the 302 participants who tested positive for other STIs, HPV, and no STIs (Table 3). Overall, most participants (245 [81.1%]) rated self-collection favorably. The most cited favorable aspects of self-collection were convenience (125 [43.6%]) and that kits were easy to use (79 [27.5%]). Most participants reported no unfavorable aspects of self-collection (182 [63.9%]); the most common unfavorable aspect of self-collection was worry about completing self-collection correctly (26 [13.1%]). Most participants (288 [96.0%]) were willing to use self-collection in the future; willingness to self-collect in the future was similar among participants negative for other STIs (206 of 214 [97.2%]), positive for other STIs (47 of 49 [95.9%]), and positive for HPV (42 of 46 [91.3%]). Most participants preferred simultaneous testing for both HPV and other STIs in future self-collection (130 [84.4%]).

**Table 3.** Perceptions of Self-Collection Among Participants With Positive Laboratory Results for Sexually Transmitted Infections From Follow-up Questionnaire

Participant perception	Participants, No. (%)			
	Overall (n = 302)	Positive for other STI (n = 49)^a^	Positive for HPV (n = 46)	No STI (n = 214)
Perception of self-collection (n = 302)				
Mostly positive	245 (81.1)	35 (71.4)	31 (71.4)	184 (86.0)
Neutral	51 (23.6)	13 (26.5)	14 (26.5)	26 (12.2)
Mostly negative	6 (2.1)	1 (2.0)	1 (2.2)	4 (1.9)
Most favorable aspect of self-collection (n = 287)				
Convenient	125 (43.6)	16 (33.3)	12 (26.7)	97 (48.37)
Easy to use	79 (27.5)	16 (33.3)	16 (35.6)	51 (25.4)
Quick results	1 (0.3)	1 (2.1)	0 (0.0)	0 (0.0)
Privacy or less embarrassing	44 (15.3)	5 (10.4)	8 (17.8)	32 (15.9)
No favorable aspects	19 (6.6)	5 (10.4)	4 (8.9)	10 (5.0)
Other^b^	19 (6.6)	5 (10.4)	5 (11.1)	11 (5.5)
Least favorable aspect of self-collection (n = 285)				
No unfavorable aspects	182 (63.9)	30 (62.5)	26 (57.8)	130 (65.3)
Worried about doing it right	26 (13.1)	4 (8.3)	6 (13.3)	35 (12.3)
Separating the brush head	7 (2.5)	2 (4.2)	0 (0.0)	5 (2.5)
Physically uncomfortable	33 (11.6)	6 (12.5)	6 (13.3)	22 (11.1)
Having to mail	4 (1.4)	1 (2.1)	2 (4.4)	1 (0.5)
Waiting for results	4 (1.4)	1 (2.1)	1 (2.2)	3 (1.5)
Other^c^	20 (7.0)	4 (8.3)	4 (8.8)	12 (6.0)
Willingness to use self-collection in the future (n = 300)				
No	12 (4.0)	2 (4.1)	4 (8.7)	6 (2.8)
Yes	288 (96.0)	47 (95.9)	42 (91.3)	206 (97.2)
Preferences about STI self-collection in the future (n = 154)				
Prefer both HPV and STIs	130 (84.4)	20 (83.3)	13 (72.2)	100 (86.2)
No preference	19 (12.3)	4 (16.7)	4 (22.2)	12 (10.3)
Prefer only HPV	4 (2.6)	0	0	4 (3.5)
Prefer only STIs	1 (2.0)	0	1 (5.6)	0

Abbreviations: HPV, human papillomavirus; STI, sexually transmitted infection.

^a^ Includes *Chlamydia trachomatis*, *Neisseria gonorrhea*, and *Trichomonas vaginalis* infections.

^b^ Other favorable aspects include doing it myself (n = 4), less physically uncomfortable or painful (n = 7), option for uninsured (n = 1), opportunity to get screened in general (n = 6), and less time consuming (n = 1).

^c^ Other unfavorable aspects include emotionally uncomfortable (n = 5), worried about privacy (n = 1), instructions unclear (n = 2), doing it myself (n = 4), no gloves (n = 1), tube size too small (n = 1), waiting for the test kit (n = 1), vial easy to spill (n = 1), brush appearance (n = 2), cost of test (n = 1), and STI self-test positive result (n = 1).

## Discussion

STI results obtained through mailed self-collection among a diverse sample of underscreened, low-income women in the United States found that participants tested positive for other STIs at a similar rate as HPV, each at 15.6%. HPV testing alone may have missed trichomoniasis, chlamydia, and gonorrhea infections in nearly 1 in 6 participants. Two-thirds of participants had reported follow-up care after positive test for other STI. Participants testing positive had similarly favorable perceptions of self-collection as a strategy to screen for both cervical cancer and STIs. Our study findings illustrate the potential of streamlined screening for other STIs alongside HPV to address multiple preventive care needs for women from marginalized backgrounds through self-collection.

This study expands upon prior research by showing the effectiveness of self-collection to increase cervical cancer screening adherence and STI screening as a combined intervention, rather than a siloed approach.^16,18,28^ Women from low-income, minoritized, and socially vulnerable communities are at increased risk for all STIs and experience notable social and structural barriers to clinic-based methods of screening.^40,41,42^ Our findings highlight the impact of streamlining multiple screenings to improve adherence to preventive care for high-risk populations historically under-screened for both cervical cancer and STIs. Similar to previous studies demonstrating high rates of acceptance and favorability of HPV self-collection,^43,44^ participants reported favorable perceptions of self-collection and preference for testing for both HPV and other STIs with self-collection in the future.

Although previously underutilized, self-collection for STI testing for chlamydia, gonorrhea, and trichomonas infections accelerated during shelter-in-place orders during the COVID-19 pandemic.^29,45,46^ Our findings support mailed self-collection as an implementation strategy for multiple STI screening in a single collected sample; however, additional implementation research is needed. Of note, the screening interval for other STIs is generally annually or more frequently based on risk,^27,47^ whereas HPV self-collection is recommended every 3 years.^48^ Implementation of streamlined screening needs to take into account different screening intervals. Another key determinant of implementation is cost to health systems and individuals. A prior cost-effectiveness analysis using outcomes from this clinical trial demonstrated that mailed HPV self-collection was cost-effective to increase cervical cancer screening uptake from a payer perspective.^49^ Other modeling studies suggest that multiple STI screening for *C trachomatis*, *N gonorrheae*, *T vaginalis*, and *Mycoplasma genitalium* is cost-effective,^50^ which may support future implementation. Additional considerations include US Food and Drug Administration (FDA) approval and insurance coverage for STI and HPV screening through self-collection. Currently, FDA approval for HPV self-collection is generally limited to in-clinic use or one at-home device, which is currently marketed at $100 or higher, depending on insurance coverage.^48,51,52^ This year, the FDA approved the first at-home STI self-test for chlamydia, gonorrhea, and trichomoniasis for direct purchase without a prescription.^53^ Market research suggests that US adults would pay out of pocket for STI self-testing in retail settings,^54^ which could inform future implementation strategies. Nonetheless, low-cost strategies, covered by insurance, are essential to reach women currently underscreened for cervical cancer and STIs, who were the focus of this randomized clinical trial.

The streamlined detection of other STIs demonstrated in this study has important implications for public health. Nonviral STIs have increased in incidence over the past decade.^6,7^ Public health experts have called for increased point-of-care testing to increase STI testing among underscreened populations.^15,55,56^ While often asymptomatic among women, STIs can increase risk for long-term sequelae from chronic infection including pelvic inflammatory disease, infertility, and pregnancy complications.^15,57^ Screening through self-collection as shown in this study can improve detection and prevent such future complications. Importantly, studies suggest that chronic cervicitis from STIs, particularly chlamydia and trichomoniasis,^35,58,59^ may increase risk for cervical dysplasia or cancer as HPV cofactors, highlighting the importance of streamlined screening for both STIs and cervical cancer prevention.

Risk factors for positivity for other STIs included non-Hispanic Black race and ethnicity, increased number of sexual partners, and smoking, which are all established risk factors for HPV infection, cervical cancer, and other STIs.^11,13^ Race and ethnicity were explored in the risk factor analysis as an indicator of the experience of racism and structural inequities in sexual health. Longstanding disparities persist in STIs, particularly among Black women.^60,61^ To date, interventions to address racial disparities in sexual health have focused on patient education and behavioral interventions.^62,63^ Our study showed that mailed self-collection detected other STIs among 51 women, the majority of whom identified as non-Hispanic Black, which may not have been detected by existing care delivery models due to barriers to care. In the randomized clinical trial, the mailed self-collection intervention had similar intervention effect on cervical cancer screening uptake when stratified by race or ethnicity.^31^ Findings from this study suggest that population health innovations such as mailed self-collection are an important tool to reach structurally marginalized groups to address persistent inequities in STIs, including the Black women who screened positive through self-collection and received treatment in this study.

As self-collection for HPV testing increases, the importance of timely follow-up of positive results is essential to ensure complete screening and diagnostic resolution and/or treatment. In our study, participants who tested positive for other STIs, regardless of HPV test result, had a higher rate of follow-up care (67%) than follow-up rate after positive HPV test from self-collection previously reported in this trial (42%).^31^ Nonetheless, this follow-up rate is lower than previously reported treatment rates for traditional clinic-based STI screening, which were 85% to 89%.^64,65^ Although self-collection has the potential to reach individuals outside of health care settings, additional investigation is needed to improve follow-up and ensure appropriate treatment of STIs detected, particularly among socially vulnerable populations. Follow-up pathways after STI positive results and HPV positive results are distinct; however, barriers to follow-up may be similar. Barriers to follow-up after positive clinician-collected Papanicolaou and HPV tests include cost, insurance coverage, time, and health literacy.^3,66,67^ These barriers have been longstanding challenges that are also now seen with HPV self-collection^68^ and are likely to also affect STI treatment and test-of-cure after positive self-collection. Additional research is needed to test interventions such as patient navigation^69,70^ to address these barriers and improve the management of positive STI tests for both HPV and other STIs. Streamlined testing for HPV and other STIs may optimize convenience by meeting multiple health needs at once and increase motivation for attending clinic-based appointments as patient interest in managing one STI may support management of another.

### Limitations

This study has limitations, including selection bias of participants who choose to enroll in randomized clinical trials, which may not be representative of the general population. The risk factor analysis was limited by small sample sizes of certain subgroups, including participants who identified as Hispanic ethnicity or an additional racial group. Outcomes included participant self-reported data, including STI treatment after positive test. Due to the nature of the trial, objective clinical data regarding STI treatment was not collected nor was treatment offered by the study team.^30,31^ Self-reported STI treatment may be subject to recall bias and increased risk for missingness due to telephone-based survey collection. Importantly, 22.1% of mailed self-collection kits were not returned, and 14 participants who returned kits did not have valid STI or HPV results. Given that our analyses were limited to participants who returned their kits and had valid results, it is possible that the STI prevalence and associated findings may not be representative of all underscreened women due to potential selection bias. Additionally, STIs tested in the intervention were limited to pathogens detectable with cervicovaginal sampling; STIs tested with serologic assays (eg, HIV, syphilis) were not included. There was variability in missing responses across questions in the follow-up questionnaire (response rate range, 47.9%-92.4%). Since the COVID-19 pandemic, self-collection has increased, as have the rates of STIs^6^; given that the trial was conducted from 2016 to 2019, prevalence of STIs may not generalize to current trends. Additionally, data regarding clinic-based STI testing was not collected; thus, this single-group analysis was limited to laboratory data from intervention participants who completed a self-collection kit. No control group participants were included, and these findings do not provide an intervention effect of mailed self-collection on STI screening.

## Conclusions

In this single-group secondary analysis of a randomized clinical trial with 327 participants, testing for both STIs and HPV in a mailed self-collection intervention detected STIs in nearly 1 in 6 participants, of whom two-thirds received follow-up care. Simultaneous self-collection for STI and HPV testing was viewed favorably. These findings highlight the importance of streamlined interventions to improve the simultaneous screening for cervical cancer and STIs and represent a novel approach to sexual health and preventive care among women of marginalized backgrounds. Future efforts should explore strategies to improve follow-up after positive screening for both HPV and other STIs.

## Notes

Supplement 1.
Trial Protocol

Supplement 2. **eFigure 1.** Study Flow Diagram **eFigure 2.** Sexually Transmitted Infections Among Participants With Positive Tests From Mailed Self-Collection Kits **eMethods. **Participant Mailed Information and Instructions

Supplement 3.
Data Sharing Statement
