# Supplementary material for: Streamlined Self-Collection Screening for Sexually Transmitted Infections and Human Papillomavirus: A Single-Group Secondary Analysis of a Randomized Clinical Trial
Source: JAMA Netw Open. 2026 Jan 8;9(1):e2551345. doi: 10.1001/jamanetworkopen.2025.51345 (PMC12784235; doi:10.1001/jamanetworkopen.2025.51345)
Supplement: Supplement 1. — Trial Protocol [file jamanetwopen-e2551345-s001.pdf]

A. SIGNIFICANCE

Early detection and treatment of cervical precancerous lesions can dramatically reduce the incidence of invasive cervical cancer (ICC).[1] The US Preventive Services Task Force (USPSTF) recommends Pap testing (cytology) alone every 3 years for women ages 21 to 65.[3,4] In 2012, the USPSTF gave an "A" rating for the testing of high-risk human papillomavirus (hrHPV) infection with cytology (*co-testing*) for women ages 30-65 to increase screening sensitivity (allowing for extension of testing intervals to 5 years).[3] HPV infection is the main cause of cervical cancer, and persistent HPV infection is consistently associated with a higher future risk of high grade precancer and cancer.[83] In April 2014, the FDA approved primary HPV physician screening for US women 25 years and older, and revisions to national screening guidelines are currently under consideration.[138]

Cervical cancer screening coverage is inadequate. *Insufficient screening is the largest factor in reducing ICC: an estimated 56% of incident ICC is due to insufficient screening, 32% due to detection failure and 13% to follow-up failure.*[131,132] According to Behavioral Risk Factor Surveillance System (BRFSS) data, more than one fifth of eligible US women report not having completed a Pap test within the last three years[4], the maximum interval recommended for Pap testing alone by national organizations[24], *yet real rates are likely higher. Comparison with medical records finds actual cervical cancer screening rates to be 10 percentage points lower than those self-reported in white women, and 26 percentage points lower in black women*.[121]

Cervical cancer is a cancer of disparities, with higher incidence rates among black, Hispanic and Asian women as compared to white women.[120] Low socioeconomic status is associated with lower screening rates and may limit women's ability to access preventive care due to resource-related barriers (e.g., poor access to services, inadequate transportation, and inflexible work hours).[25] Significant progress has been made in recent decades to reduce ICC incidence in developed countries, but expanding benefits of screening to medically under-served US women remains a challenge. The Affordable Care Act (ACA) has the potential to increase cervical cancer screening rates by expanding insurance coverage and requiring the provision of free screening services. However, access barriers remain even after implementation of ACA, since Medicaid expansion has not been implemented in all states,[26,27] and only an estimated 63% of Medicaid-eligible adults participate in Medicaid.[28] *Because low-income uninsured and publically insured women are at a higher risk of being under-screened, research is needed that focuses on this population.*

Phone-based "enhanced reminders" to complete screening are effective in increasing cervical cancer screening uptake. Recent reviews from the Community Preventive Services Task Force (CPSTF) and Cochrane Collaboration recommend client reminders as an effective method for increasing in-clinic cervical cancer screening compared to usual care among both regularly and under-screened women (median increase 10%; RR 1.44 [95% CI: 1.24,1.67]).[19,20] *RCTs and population-level studies from the US and Sweden show that providing screening reminders with appointment scheduling assistance ("enhanced reminders") is an effective method to increase in-clinic screening uptake*, although it does not get all women into screening.[21,22,29,30]

Mailed at-home HPV self-collection has the potential to increase cervical cancer screening completion among under- and never-screened women. HPV self-collection is a technique by which women use a simple collection device to obtain cervico-vaginal cell samples to test for infection with high-risk (oncogenic) HPV infection, an objective indicator of women's elevated risk for cervical cancer. Self-collection for HPV testing compares favorably in sensitivity and specificity to that of physician-collection for the detection of HPV infection and of high-grade cervical lesions.[5,6-8,31-36] Our pilot studies in North Carolina (NC) show that at-home HPV self-collection kits delivered via mailing are highly acceptable to under-screened, low-income women (69 to 85% return rates),[37,38,47] consistent with high HPV self-collection acceptability demonstrated in other states[39-42,44] and countries.[45,46,48-51]

To our knowledge, only one US study has evaluated the effect of at-home HPV self-collection on screening uptake, distributing kits via door-to-door recruitment.[39] HPV self-collection kits were offered to women to complete immediately and return directly to the recruiter. This study assessed only completion of HPV self-collection as screening completion, and did not assess follow up to in-clinic Pap smear screening among self-collection HPV-positive women. Other US studies investigating self-collection have focused on acceptability or validation of self-collected samples versus physician-collected samples, rather than the effect of HPV self-collection on in-clinic screening uptake.[32-38,40-44,84-90,92-100]

Several European studies have found that offering at-home HPV self-collection to under- and never-screened women via direct mailing leads to higher screening completion compared to mailed reminders (e.g., 30.8% via self-collection vs. 6.5% via reminders in the Netherlands, $p<0.001$[12]).[9-16,52] These studies found relatively high rates of follow-up to in-clinic Pap testing among women receiving HPV positive self-collection

results (e.g., 86% in another Dutch study[16]),[9] and that use of at-home HPV self-collection led to higher detection of CIN-2+ (cervical intraepithelial neoplasia: high grade precancerous lesions) than mailed written reminders alone.[10,12,13,15,16] *While research is promising, all effectiveness studies were conducted in countries with national screening registries and universal health care, neither of which exists in the US. Domestic studies are novel and necessary to evaluate effectiveness in the context of the US healthcare system.*

Beliefs and emotions regarding cervical cancer and screening motivate screening uptake. Behavioral theory and empirical evidence indicate that lower levels of perceived barriers and higher levels of perceived risk of developing cancer are associated with higher screening completion.[17,18,57,58] The effect of increased perceived risk on increasing preventive action is particularly strong when people are provided with a clear and immediate course of action to alleviate their risk, such as an offer for free follow-up screening.[59,60,121] Substantial evidence already exists that women perceive lower barriers to self-collection than to in-clinic screening.[40,42,44,45,46,51] In the proposed trial, we will assess the novel question of how self-collection affects perceived risk of cervical cancer, and its role as a mediator in increasing in-clinic screening.

Limited research suggests that HPV self-collection would be cost-effective. A model evaluating at-home HPV self-collection to triage HPV-positive women to follow-up colposcopy found this approach to have slightly lower lifetime cost than cytology-based (Pap) screening.[84] This US study was based on national cytology screening uptake rates (80%), and on HPV self-collection uptake in a sample of women who had recently received in-clinic cervical cancer screening, rather than in a higher risk under-screened population.

HPV self-collection has the potential for widespread application and impact on public health.

If at-home self-collection is found to be effective at increasing cervical cancer screening completion among under- and never-screened US women, there are multiple pathways for its future implementation. Clinics could undertake outreach programs by distributing kits to under- and never-screened women via direct mail, at clinics that do not provide screening (e.g., flu or STD), or other public events, similar to current use of Fecal Immunochemical Tests for colorectal cancer screening.[143,144] The NC Breast and Cervical Cancer Control Program (NC BCCCP) has expressed interest in using the self-collection method if it is found effective in increasing screening among women who are failing to attend in-clinic screenings (see LOS). Self-collection kits could be distributed via walk-in medical clinics located in some pharmacies and box stores for completion at home or immediately in the store restroom. There is also potential for phone-based dissemination of the self-collection kits. Each year, state and national surveys identify large numbers of women overdue for screening by national guidelines, yet these surveys have no options for a "next step" to offer screening to these women. Additionally, a variety of hotlines provide services to women potentially at high risk of being under-screened. United Way 2-1-1 social assistance hotlines and the American Sexual Health Association (ASHA) call center are interested in implementing HPV self-collection outreach if the intervention is found to be effective (see LOS). Based on study findings, we will seek extramural funding from federal and private agencies to scale up the intervention to state and national platforms.

The under- and never-screened women targeted by the proposed study are those at highest risk for developing cervical cancer. As a new approach to reaching these high-risk women, this intervention has the potential to have a meaningful impact on ICC incidence and mortality. The method will have even greater potential impact if approved by the FDA for primary screening. Approval would allow for referral of only self-collection HPV positive women to follow-up in-clinic screening, with women with negative self-collection results considered screening complete. This approach would be particularly beneficial for women who have trouble regularly accessing clinics. Improved understanding of the psychological factors that mediate the relationship between HPV self-collection and subsequent in-clinic screening will provide insight into possible ways to maximize the intervention's effect.

By collecting cost data associated with the intervention relative to the control arm, we will be able to provide important information to state and national decision-makers about resource requirements to implement the self-collection intervention. Such data are particularly meaningful in fixed resource settings, such as those serving uninsured and Medicaid-insured women. Because state and safety net health budgets are usually limited by fixed resources, assessing value for money is critical. Beyond scope of this grant, economic data obtained will provide the basis for future, more sophisticated cost-effectiveness analyses (for example, considering longer term cervical cancer mortality implications through simulation) and budget impact analyses for policy decision makers and payers. Given the measurable benefits of early and regular cervical cancer screening on cancer outcomes, including cervical cancer mortality, the tradeoffs in terms of health value and financial resources should be considered in any comprehensive cancer screening intervention assessment.

B. INNOVATION

At-home HPV self-collection is a novel methodology that takes advantage of developments made in the past decade with i) brush designs that are minimally invasive, well-accepted, and effective for self-collection; ii) sample preservation media that is non-toxic, safe for mailing, and stable at a range of temperatures; and iii) well-validated, field-tested illustrated instructions for self-collection that are comprehensible to low-literacy women. *Mailing self-collection kits, as opposed to a clinic-based or face-to-face distribution has the potential to reach a much broader segment of the medically underserved US population.* Our recent research conducted in NC found that mailing kits for HPV self-collection is well-accepted and feasible for use with low-income, under-screened women (see Preliminary work).[37,137] This proposed RO1 study builds on these findings to examine whether this approach is effective in increasing completion of cervical cancer screening within this high-risk population (Aim 1), potential mechanisms explaining the intervention's effect or lack of an effect (Aim 2), and estimated incremental costs of the intervention (Aim 3).

No US studies, to our knowledge, have assessed the effectiveness of mailed HPV self-collection kits to increase completion of cervical cancer screening with follow-up to either in-clinic screening among HPV-positive women, or investigated its impact on the detection of high-grade cervical intra-epithelial neoplasia (CIN-2+). Follow-up to in-clinic screening among HPV positive women is a key measure of intervention effect, because prevention of ICC depends on subsequent screening (cytology and histology) to obtain specificity necessary for CIN-2+ confirmation and treatment.[8] *Published studies have also not examined psychological mediators or assessed the incremental costs of the mailed self-collection intervention, both investigations which we propose in the current study. Though studies outside the US have shown that mailing kits for at-home HPV self-collection is effective in increasing cervical cancer screening uptake among under- and never screened women, it has not been investigated in the context of the US healthcare system. Implementation is likely to notably differ in the US system, which lacks national screening registries and universal healthcare*.

C. APPROACH

Preliminary work: Drs. Smith and Brewer have collaborated on two successful pilot studies using mailed, at-home HPV self-collection among low-income, under-screened NC women. The first study, *My Body, My Test-1*, assessed feasibility and acceptability of at-home HPV self-collection via mailed kits. Eligibility criteria were the same as for this proposed RO1 study, except that private insurance was permitted. Of 481 eligible high-risk women recruited, 339 successfully returned the kits (70% return kit rate).[37] Most study participants were African American (57%), without private insurance (64%), and with household income below 200% of the federal poverty line. Median participant age was 43. *Women reported high satisfaction (83% positive or neutral) with the self-collection experience, citing convenience, privacy, and ease of use.* Of returned samples, 15% were HPV DNA positive and only 2.5% were inconclusive by Hybrid-Capture II testing (QIAGEN, Gaithersburg, Maryland). As well as providing evidence on acceptability, this pilot study allowed our team to refine methods for recruitment, sample processing, data management, and participant retention. We found that facilitating follow-up to in-clinic screening and developing collaboration with clinic partners was critical to maximize in-clinic screening uptake.

The second study, *My Body, My Test-2,* was designed to compare results of mailed, at-home HPV self-collected samples to results from (a) in-clinic self-collected HPV samples, (b) physician-collected HPV samples, and (c) liquid-based cytology (Pap). This study was completed in May 2014. Eligibility criteria were identical to the current proposal. *HPV RNA positivity was 13.6% using the Aptima HPV assay (Hologic, San Diego, California) among women who returned at-home self-collected samples.*[137] *We found fair agreement between at-home and physician-collected samples (Kappa 0.71, 95% CI 0.53-0.89). Of women completing in-clinic screening, 11.5% presented with cervical abnormalities of ASCUS+ and 2.8% with histologically confirmed CIN2+ (notably higher than in the general US population).*[38,137] *All CIN2+ cases tested positive for high-risk HPV in both home and clinician samples. Most participants returned home self-collected samples (83.1%) and completed clinic appointments (76.9%).* This second study allowed our team to refine recruitment methods, including the use of local recruiters and collaboration with community clinics. We have developed and refined protocols for appointment scheduling coordination between the study call center and study clinics, efficient sample return and processing, and managing laboratory testing and results in accordance with standards for diagnostic use.

This preliminary work provides valuable data on the acceptability, feasibility, and validity of mailing kits for at-home HPV self-collection to under-screened NC women. *However, both were one-group studies, with no assessment of the effectiveness of HPV self-collection for increasing in-clinic cervical cancer screening, as proposed here.*

Dr. Smith has considerable experience in the completion of HPV collection studies domestically and internationally. Prior studies include an RCT examining the effect of male circumcision on natural history of HPV infection among men, in collaboration with Dr. Hudgens (Co-I on the proposed study).[54,55] In a sample of 2,299 men followed over 24 months, male circumcision reduced HPV acquisition and reinfection and increased HPV clearance in the glans site for both incident and prevalent penile HPV infections.[126] This prior RCT demonstrates Dr. Smith's and Hudgens's experience successfully implementing a large field-based RCT intervention study and completing associated manuscripts for publication.

Figure 1. Study flowchart

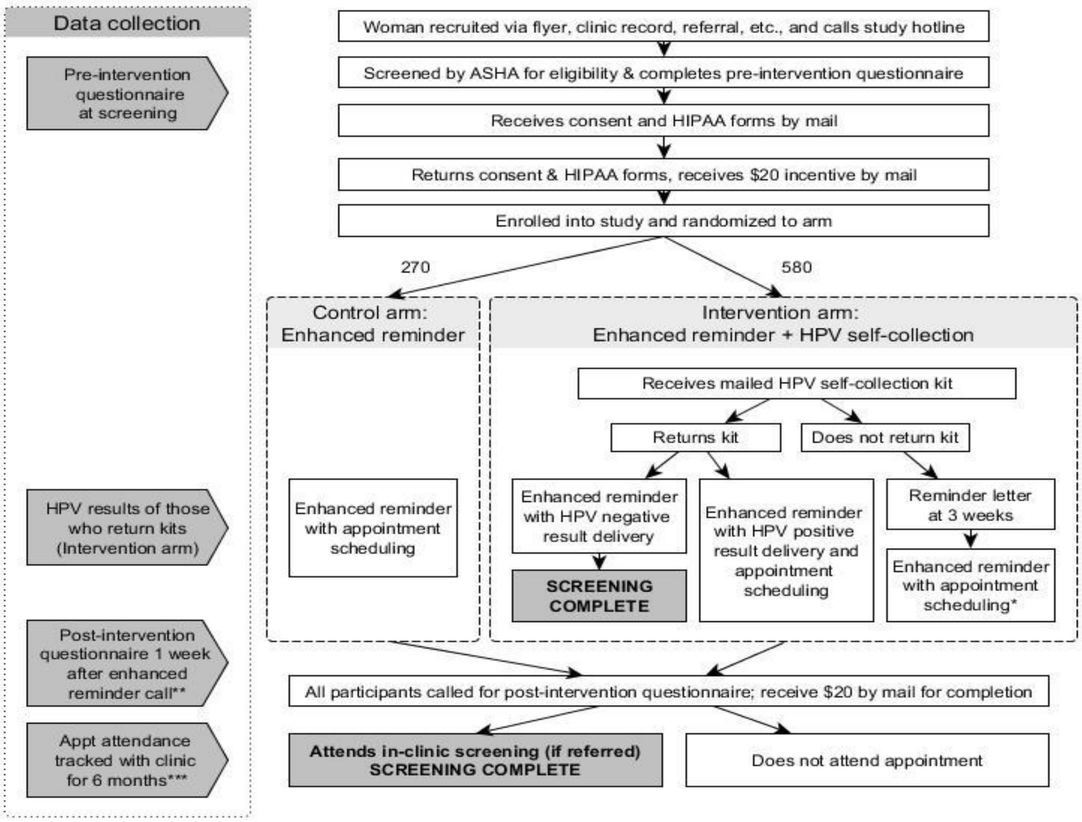

*Intervention arm participants who have not returned their kit 2 weeks after reminder letter will be called to schedule an appointment. **Control arm participants will be contacted for post-intervention questionnaire if they have not scheduled an appointment within 5 weeks of enrollment. ***HIPAA authorization will allow for tracking of appointment attendance directly with clinics. Participants who have not attended a study clinic within 6 months of enrollment will be contacted to see if they attended another clinic. Participants with abnormal results will be tracked to ensure follow-up screening and treatment.

Overview of the experimental design. The proposed study is a two-arm RCT examining the effect of HPV self-collection with enhanced screening reminder compared to enhanced reminder alone (Figure 1). Potential participants will call the study hotline to be screened for study eligibility. If eligible, women will complete a pre-intervention questionnaire during this call to collect demographic data and behavioral measures for use in mediation analysis (Specific Aim 2b). Women will be considered enrolled when they return informed consent and Heath Information Portability and Accountability Act (HIPAA) forms authorizing the study to obtain in-clinic cervical screening–related medical records. Participants will then be randomly assigned to the control or intervention arm. *Both arms will receive an enhanced reminder phone call: education on cervical cancer screening and assistance scheduling an appointment for free screening at a study-affiliated community clinic, if needed.* The control arm will receive only the enhanced reminder call. The intervention arm will also receive kits to self-collect cervico-vaginal samples and be asked to return the samples by mail for oncogenic (high-risk) HPV testing. If a participant returns her kit, she will receive her self-collection HPV results during the enhanced reminder call (See 1b). Self-collection HPV positive women will also receive assistance scheduling during the enhanced reminder call. *HPV negative women will be considered screening complete and will not be scheduled for in-clinic screening*. Women who do not return self-collection samples ("non-returners"), will receive the enhanced reminder call with scheduling assistance. *Screening completion will be defined as attending in-clinic screening or receiving a negative HPV self-collection result.* One week after the enhanced reminder, all participants will be called to complete a post-intervention questionnaire. For all women referred to in-clinic screening, clinic attendance, screening results, and follow-up screening and treatment will be tracked directly *with clinics* for analysis and to ensure appropriate care.

Conceptual model. Our conceptual model for the intervention effect centers around perceived risk, a construct from the Health Belief Model (HBM).[58] We will assess the mediational role of perceived risk in the relationship between the self-collection intervention and in-clinic screening.[78,79] High-risk HPV results are an objective indicator of women's risk for cervical cancer. Evidence suggests that an increase in *perceived risk* of

cancer increases intention and action towards cancer screening.[17,101] Perceived risk has a cognitive aspect that we operationalize as *perceived likelihood* of developing cervical cancer and an affective (or emotional) aspect that we operationalize as *worry*. Our model also includes an exploratory risk construct, *embodiment of risk,* which we operationalize as the sense of awareness of and connection with one's body as a source of health and risk.[56,76] We anticipate that women who complete at-home HPV self-collection and receive HPV positive results will experience an increase in perceived risk and thus be more likely to complete subsequent in-clinic cervical cancer screening. *Though elevated worry can result in maladaptive "fear control" responses (such as denial or deciding to not think about the threat), this effect occurs most strongly with low-efficacy messaging: if the individual believes herself incapable of taking preventive action (low self-efficacy), or if she does not believe the action will reduce risk (low response efficacy). However, higher perceived risk with high-efficacy messaging has been shown to increase adaptive preventative behavior.[59] We will deliver HPV results along with an offer for free follow up screening (supporting increased self-efficacy), and with education that cervical cancer is highly treatable when detected early (increasing perceived response efficacy).* We expect that HPV negative women, women in the control arm, and non-returners will also experience some increase in perceived risk after receiving basic education about cervical cancer provided to all participants during the enhanced reminder call, but this effect is expected to be similar between these groups. The model does not include other HBM constructs, though we will measure these constructs as potential mediators. We do not expect perceived severity of cervical cancer or perceived effectiveness of screening to vary between arms, as education regarding these topics will be identical between arms. We expect that any increase in self-efficacy to complete in-clinic screening will come from the enhanced reminder, which is present in both arms. Cues to action, though part of the HBM, we consider to be an environmental factor (e.g., a doctor's recommendation). In this study, the intervention itself is the cue to action, and thus external to the mediation analysis.[18]

Figure 2. Conceptual model

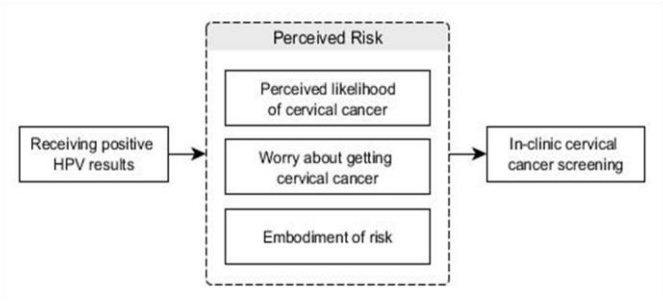

AIM 1. Determine whether at-home HPV self-collection increases the completion of cervical cancer screening among under-screened women receiving enhanced reminders.

1a. Recruit 870 under- or never screened, low-income women ages 30 to 64

Eligibility requirements and county selection. Participants will be 870 women under- or never-screened for cervical cancer, ages 30 to 64, uninsured or on public Medicaid insurance, non-pregnant, without a history of hysterectomy, English-speaking, and ≤250% of the poverty line. We define *under-screened* as without self-reported Pap screening in 4 years or more and with no history of HPV/Pap co-testing. HPV testing is recommended for women 30-65, thus, we set a lower limit of age 30. Study income, age, and insurance eligibility criteria were selected to ensure that participating women are eligible for free clinic appointments and follow-up care through the NC BCCCP program or Medicaid.

We selected 4 NC study counties based on high cervical cancer burden (Table 1). ICC incidence and mortality rates are highest in the state's lowest resource counties.[62] We selected Mecklenburg, the most populous NC county, along with Cumberland and Robeson, two counties with particularly high rates of ICC and poverty, to ensure sufficient numbers of low-income, under-screened women eligible for recruitment, as well as a sufficient African American population to provide a racially diverse sample. We selected Buncombe County based on a large number of women in our age range, excellent past collaboration, and particularly strong capacity to conduct chart review and referrals for recruitment of underscreened women.

Table 1. *Target county profiles (2010 Census data unless otherwise noted[63])*

Cervical cancer, 1998-2007[62]					Women aged 30-64	Poverty rate	Uninsured	Un-screened in 3+ years[4]	Race	
	Incidence		Mortality						Black	White
	N	rate*	N	rate*	N	%	%	%	%	%
North Carolina	*3,652*	*8.2*	*1,208*	*2.6*	*2,289,563*	*15.5*	*16.2*	*18.4*	*22.0*	*72.1*
Cumberland	**144**	10.0	54	**4.1**	71,818	16.6	14.2	21.2	**36.9**	54.6
Mecklenburg	**278**	7.2	75	2.1	**227,703**	12.5	16.8	18.8	**31.5**	60.7
Robeson	79	**12.8**	33	**5.2**	30,760	**30.2**	23.8	21.4	**24.9**	33.1**

Buncombe	77	6.4	31	2.3	**114,996**	**16.8**	**16.5**	17.4	6.4	87.4

**per 100,000 women over a 10 year period.* **Approximately 1/3 (38%) of the Robeson County population is American Indian

Recruitment methods. Acknowledging the challenges of recruiting a medically underserved population, we will draw on our considerable experience recruiting this population in previous pilot studies (See Preliminary work). For the proposed study, we have obtained support from health departments, social services, and community organizations to reach potentially eligible women (LOS). We have established partnership with United Way's 2-1-1 social assistance hotline to offer study information at the end of regular calls, an approach that led to successful recruitment of hundreds of women in pilot studies. We will work with United Way and service providers to develop comprehensive lists of agencies and organizations providing services to low-income women in study counties. These groups have previously been local advocates for our recruitment efforts, distributing materials to clients and hosting recruitment events. Given that word-of-mouth was one of the most effective recruitment tools in our pilots, we will ask active and potential participants to tell friends and family about the study. We will also work with partner clinics to conduct medical records review to identify patients overdue for screening, as done in our pilot studies.

Population sampling will be non-probability, based on women who respond to recruitment efforts. While this approach may oversample more motivated women and somewhat limit the study's generalizability, recruitment from the general population allows for outreach to medically underserved women who do not regularly utilize clinic services. Because under-screened women comprise ~one fifth of the total population, it is not logistically feasible to randomly sample from the general population. By partnering with agencies in study counties, focusing on four NC counties with large numbers of under- and unscreened women, utilizing our considerable experience in recruiting this population, and hiring local coordinators to assist in each county, *we will be able to successfully complete recruitment and enrollment of 870 study-eligible women, equaling an average 24 women per month (~6 per month for each county) over 3 years.*

Screening and enrollment. Women who learn about the study through outreach efforts will call a toll-free study hotline run by the American Sexual Health Association (ASHA). The ASHA call center has provided 24-7 hotline services for our pilot studies. Call center agents will screen callers for eligibility criteria. Eligible women will be verbally consented to complete a pre-intervention questionnaire during this eligibility call to obtain socio-demographic data and behavioral measures to compare with post-intervention measures (Aim 2b). Agents will collect contact information (e.g., multiple phone numbers, mailing addresses, and emails) to provide multiple options for follow-up. Eligible women will be mailed an informed consent form and a HIPAA authorization that allows the study to obtain clinic screening and treatment results. To enroll, women must return both signed forms in a pre-paid, pre-addressed envelope. They will receive $20 by mail for form return.

1b. Randomize participants to receive either mailed kits for at-home HPV self-collection with enhanced reminders (intervention arm) or enhanced reminders alone (control arm)

Randomization. Enrolled women will be randomized to the control or intervention arm. Twice as many participants will be randomized to the intervention arm as to the control arm in order to provide sufficient power for Aim 2 analyses. A permuted block design will be employed using blocks of size 9 (6:3 intervention: control). Randomization will be stratified by county to ensure that participants are in equal proportions in each arm.

Control arm procedures (enhanced reminder only). *Participants in both control and intervention arms will receive an enhanced reminder: a phone call providing (i) education on cervical cancer, and (ii) assistance scheduling an appointment for free screening at a study-affiliated clinic if needed. Education will address that (i) cervical cancer is highly treatable when detected early; (ii) the participant is due for screening based on doctor recommendations, and (iii) the participant is eligible for free screening in her community. Appointment scheduling will be completed during the enhanced reminder call.* As in pilot studies, we will establish protocols with study clinics that allow the call center to schedule appointments with participants directly. Control arm participants will receive the enhanced reminder call one week after enrollment, the same approximate time between enrollment and receipt of the self-collection kit in the intervention arm. If participants choose not to schedule at this time, they will be provided with the phone number of the local study clinic to call when they choose. Agents will make 3 call attempts to reach participants, leaving a message each time, if possible.

Intervention arm procedures (*enhanced reminders plus HPV self-collection*). Women in the intervention arm will also receive a self-collection kit by mail with instructions on how to self-collect a cervico-vaginal sample and return it by mail for HPV testing. The self-collection kit will contain a brush and vial of sample preservation solution; simple illustrated instructions; a toll-free number to call with questions; and pre-addressed, pre-paid mailers for sample return. Samples will be tested at Labcorp for high-risk HPV infection (details below), with an average turn-around time of 1 to 2 weeks. In pilot studies, average time between

mailing kits to participants and receiving completed samples was 3 weeks, so we estimate that we will be able to call participants with their HPV results at 4 to 5 weeks after enrollment. Delivering HPV results by phone has been well-accepted by participants in pilot studies (96% of participants were "comfortable getting self-test results by phone"[37]) *and allows for immediate education and counseling to reduce any potential anxiety.*[139] *Scripts for delivering HPV results will be carefully developed with ASHA to address common concerns and points of confusion identified by research on cervical cancer education.*[128-130] *Messages will be pilot tested with women from the target population to assess comprehension and assess efficacy in addressing potential anxiety or fear responses (see Conceptual model).* ASHA staff is well-trained to counsel women experiencing anxiety about results, and will refer participants to additional clinical counseling if needed.

At the time of enhanced reminder/HPV result delivery call, HPV positive women will have the opportunity to schedule a free appointment for in-clinic screening (as described in the control arm above). *Because HPV self-collection is not yet approved as a primary diagnostic test in the US, HPV negative women will be told that though their results indicate that they are at lower risk for cervical cancer, these results are not conclusive and that they are overdue for in-clinic screening (messages will be pilot tested as described above). We will provide HPV negative women with the contact information of the study clinic, where they may receive free in-clinic screening if they choose. We will not actively provide scheduling assistance to HPV negative women during the enhanced reminder call. Analysis to assess the intervention effect will consider all women with HPV negative self-collection results to be screening complete, as they would be in real-world implementation (see Aim 1a).* A participant who has not returned a self-collected sample within 3 weeks will receive a mailed reminder letter to return the kit. If the study has still not received her sample 2 weeks later, we will call her to provide the enhanced reminder call and offer to schedule in-clinic screening. The study will make 3 call attempts to all participants to complete the enhanced reminder. If these attempts fail, the participant will be sent a letter inviting her to call the study hotline to receive her HPV results and/or schedule an appointment.

In-clinic screening tracking. *We will track in-clinic screening completion for all study participants for 6 months directly with the clinic using clinic medical records, as permitted by HIPAA authorizations obtained at study enrollment.* Participants who do not attend an appointment at a study-associated clinic during this time period will be contacted by phone (6 call attempts) to ask if they obtained in-clinic screening at another clinic. If unable to reach the participant, we will follow up with other clinics in the county and neighboring counties to determine if the participant attended a screening appointment at another location in the preceding 6 months. Because the receipt of study incentives is not linked to clinic attendance, screening completion should not be affected by financial motivations.

Post-intervention questionnaire. We will call participants to complete a post-intervention questionnaire by phone one week after completion of the enhanced reminder call (or 3 failed attempts). This interval was selected so that the participant will complete the questionnaire after the intervention and before the clinic appointment, in case the completion of in-clinic screening affects the behavioral predictors of interest. Participants will be able to complete the questionnaire at the clinic if they have not already done so. Questionnaire data collected at the clinic will be compared to data collected from participants pre-appointment to look for differences, and will not be included in mediation analysis in case in-clinic screening has affected key measures. Post-intervention questionnaire items will match those on the pre-intervention questionnaire to assess change (Aim 2). The questionnaire will also collect basic process measures, such as acceptance of the self-collection and enhanced reminder call process. Participants will receive $20 for questionnaire completion.

Participant retention. In pilot studies, we achieved high rates (82%) of follow-up with participants.[37] We will use the same protocol for questionnaire completion: 6 calls made over 2 weeks at different times of day, using multiple phone numbers collected at recruitment, including the number of someone who can reach the participant if her phone is not working. We use email if an address is available. If we are unable to complete the post-intervention questionnaire by phone or email, we will send the participant a final follow-up letter.

Sample self-collection (intervention arm only). Self-collected samples will be collected using a Viba brush (Rovers Medical Devices, B.V., The Netherlands) and preserved in Aptima sample transport medium (Hologic, San Diego, CA). In validation studies, self-collection using the Viba brush had substantial agreement with physician cervical sample collection (e.g., Kappa 0.70 [95% CI: 0.60-0.78])[64-66]. The sample transport medium is non-toxic, approved for mailing via US Postal Service, and validated to keep specimens stable for HPV DNA and RNA testing up to 60 days at room temperature.[67,68] Illustrated instructions for completing self-collection were developed for comprehension in low-literacy populations using NIH plain language guidelines and evaluated in pilot study focus groups with the target population (See Appendix).[69]

In-clinic sample collection. *We will work with one clinical partner in each of the 4 counties to provide co-testing or Pap alone (per participant preference) to all study participants who attend clinic appointments. Health departments in Buncombe, Cumberland, and Mecklenberg will provide screening at 2 clinics each (NC-BCCEDP and family planning). Robeson Heath Care Corporation will work in one central clinic (See Facilities).* A certified clinician will collect endo-cervical samples using the ThinPrep cervical spatula and endocervical brush or broom-like device and preserve them in PreservCyt solution (Hologic GenProbe, Bedford, MA).

Sample handling and laboratory testing. Self-collected samples will be mailed directly to UNC, where they will de-identified for delivery to the Laboratory Corporation of America Holdings (LabCorp) laboratory in Burlington, NC. Clinic samples will be mailed directly to LabCorp. Clinic and self-collected samples will be tested for 14 high-risk HPV types using the FDA-approved Cobas HPV Test (Roche, Indianapolis, IN), and if positive, with type-specific assay for HPV 16 and 18. Self-collected samples will also be tested for beta-globin positivity, a validated measure of cell sample viability, to determine whether the samples contain sufficient specimen.[70] Liquid-based cytology will be conducted using the ThinPrep 3000 processor (Hologic GenProbe, Bedford, MA). Per standard protocol, cervical samples found to have abnormal cervical cytology of ASCUS or greater will be reviewed and evaluated by the lab pathologist. Because cytology and physician-collected HPV results will be used for referral to follow-up screening, colposcopy and potential treatment, they will be reported directly to attending clinics and obtained by study staff using HIPAA authorizations. Self-collection results, as they are not yet approved for clinical use, will be reported directly to staff and participants as research results.

1c. Compare screening completion (primary outcome) between the intervention and control arms.

Measures: *Screening completion will be defined as in-clinic screening or receipt of negative HPV self-collection results. Women with HPV negative self-collection results will be considered screening complete. All other participants will be considered screening complete if they obtain co-testing or Pap smear screening at a study-affiliated or other local clinic.* Study clinics will offer co-testing and Pap alone per participant preference.

We calculated sample size required to have 80% power to detect a difference in screening completion between intervention and control arms. We assume a true difference between the arms of at least 10%, with a 2:1 intervention: control randomization ratio (Table 2). For a range of expected screening completion proportions in control and intervention arms, required sample sizes for the control arm range from 231 to 290. To ensure sufficient power, we have chosen the largest required sample size: 290 women in the control arm and 580 women in the intervention arm. Our required total sample size is thus 870.

Table 2. *Necessary sample sizes to detect differences in cervical cancer screening (by screening completion between intervention and control arms, at 0.8 power, intervention arm sample size twice that of the control.*

Uptake of in-clinic cervical cancer screening		Required sample size		
Intervention	Control	Intervention	Control	Study total
60%	50%	580	290	870
65%	55%	560	280	840
70%	60%	529	264	793
75%	65%	486	243	729
80%	70%	431	231	662

Outline of Statistical Analysis Plan (SAP).

Eligibility screening: Frequency of reasons for exclusion from study will be reported.

Pre-intervention: Demographic characteristics (e.g., age, race, marital status, education, parity, and insurance status) will be collected using items from the US Census. Distributions of pre-intervention variables will be compared between study arms to assess any chance imbalances in covariates. Continuous variables will be analyzed using the Wilcoxon rank sum test, and categorical variables with Fisher's exact test.

Primary outcome variable: The primary outcome variable will be completion of cervical cancer screening (*HPV negative by self-collection or completion of in-clinic screening with i. cotesting or ii. Pap smear alone*).

Primary analyses: Primary analyses will employ the *intent to treat* principle wherein outcomes are analyzed according to study arm regardless of whether the participants complied with their arm's intervention components. Primary analyses will entail comparing the proportion of women in each study arm that complete cervical cancer screening. The usual Z statistic for comparison of two binomial proportions will be used to assess whether there is a significant difference in the proportions between the study arms. The difference in proportions will be reported with 95% confidence intervals based on the Normal approximation to the binomial, which should be valid given the planned sample size and anticipated proportions of screening completion in each arm. The primary analysis will consider a woman referred to clinic as achieving the primary endpoint if she completes clinic based screening at any location, regardless of whether the site is a study-associated clinic*. Note that unlike typical cohort studies, loss to follow up (LTFU) is equivalent to failure to complete our primary endpoint of completing screening*.

Secondary analyses: The effect of the intervention will be assessed among the subset of study participants who were compliant with their randomization assignment and the study protocol. Appropriate methods will be used to guard against possible selection bias induced by conditioning on compliance:[71]

(1) Log-binomial regression modeling will be used to determine whether baseline demographic covariates are associated with completion of (i) at-home self-collection in the intervention arm and (ii) screening completion (as defined above).

(2) The intervention effect will be assessed using log-binomial regression modeling adjusting for baseline covariates associated with randomization (not expected) or with completion of screening.

Exploratory analyses:

(1) We will assess differential intervention effects by race, age, income, and educational level.
(2) We will compare detection rates of high-grade CIN-2+ lesions between the control and intervention arms.
(3) We will compare follow-up colposcopy and treatment, if indicated, between control and intervention arms.

AIM 2. Examine possible mechanisms explaining the intervention's effect, or lack of an effect.

2a. Compare in-clinic screening completion of women who receive positive HPV self-collection results to completion of (i) women in control arm, and (ii) women who do not return a self-collection kit.

Our primary outcome measure for aim 2a is the completion of in-clinic screening. Our exposure variable of interest is the receipt of a positive HPV self-collection result. We will compare in-clinic screening completion in women who receive a positive HPV self-collection result to in-clinic screening completion in (i) women in the intervention arm who do not return a self-collection sample ("non-returners"), and (ii) control arm participants. Our pilot studies in NC have found self-collected sample return rates of 70% and 87%. Estimated study power is presented below given enrollment of 580 participants in the intervention arm, a conservative 70% return of self-collected samples, and an estimated 15% HPV positivity in self-collected samples.

Table 3. *Estimated power to detect differences in screening completion among participants referred to in-clinic screening*

In-clinic screening uptake among HPV positives (n=61)	In-clinic screening uptake among women in control arm (n=289)		In-clinic screening uptake among women who do not return kit (n=174)	
%	%	Power to detect	%	Power to detect
65%	45%	82%	40%	90%
70%	50%	83%	45%	90%
75%	55%	85%	50%	92%
80%	60%	87%	55%	94%
85%	65%	91%	60%	96%

Given a range of potential in-clinic screening completion of 65-85% in HPV-positive women, we have sufficient power to detect a difference in screening completion between HPV positive women and (i) control arm participants given true difference of at least 20%, and (ii) non-returners given a true difference of at least 25%.

Primary analysis for Aim 2a will utilize Fisher's exact test to assess whether receiving a positive self-collection HPV result is associated with higher uptake of in-clinic screening. Exact logistic regression modeling will be used to assess the association between receipt of a positive HPV result and uptake of in-clinic screening among women referred to in-clinic screening while adjusting for baseline covariates associated with either HPV positivity or uptake of in-clinic screening. We expect that ~2.5% of self-collected samples will yield an inconclusive result; these women will be referred to in-clinic screening. We expect that an inconclusive HPV result will produce similar levels of elevated perceived risk as a positive HPV result. Therefore, primary analyses for Aim 2a will include women with inconclusive test results with HPV positive women. We will conduct a sensitivity analysis to assess whether the analysis results change if women with inconclusive test results are not included in this group.

2b. Assess perceived risk of cervical cancer and beliefs about screening via pre- and post-intervention questionnaires among all women

All participants will complete questionnaires to assess behavioral constructs at recruitment (pre-intervention) and after the enhanced reminder call (post-intervention). We will use validated and reliable survey measures from previous studies (Table 4). We will cognitively test survey measures in 2 rounds of 7-8 women from our target population, and modify items if needed.[72] Our experience is that this number of interviews is adequate to detect most problems with wording[73]; however we will add more interviews if needed.

Table 4. *Example questionnaire measures*

Construct	Item	Response scale
Perceived likelihood	Without regular screening, what do you think is the chance that sometime in the future you will get cervical cancer? [74]	No chance - high chance
Worry	How worried are you about developing cervical cancer? [75]	Not at all - extremely

Construct	Item	Response scale
Embodiment of risk	I don't really know where my cervix is.;<br>When I have a menstrual cramp, it makes me wonder if my cervix is unhealthy.[102,103,]	Strongly disagree - strongly agree
Other Health Belief Model (HBM) constructs	E.g., perceived effectiveness: How effective do you think that screening is in preventing cervical cancer?[77]	Not effective at all - very effective

2c. Examine the role of perceived risk as a psychological mediator of the intervention effect.

According to our model, perceived risk of cervical cancer should increase from pre- to post-intervention questionnaires in women who receive HPV positive self-collection results, and women with higher perceived risk should be more likely to complete in-clinic screening. The general approach for mediation analysis is to establish that the predictor variable (receipt of HPV positive result) is associated with the outcome (behavior) and mediating variables (beliefs). Then one establishes that controlling for the mediators meaningfully reduces the size of the association of the predictor variable and outcome. Analyses described in Aim 2a will have already established the first analytic step required for mediation: evaluation of the effect of receipt of HPV positive results on in-clinic screening completion among women referred to screening. Next, using the measures described in 2b, we will examine whether the components of perceived risk changed as proposed in the conceptual model. Finally, for women referred to in-clinic screening (self-collection HPV positive, non-returners, and control arm), we will examine whether changes to in-clinic screening completion remain after controlling for beliefs affected by the intervention. We will also conduct mediational analyses using other constructs from the HBM (perceived effectiveness, perceived barriers, self-efficacy, and perceived severity). We will examine each belief separately as a potential mediator, controlling for variables identified as associated with randomization assignment. Analyses will use the MacKinnon method to compare coefficients for the intervention-behavior pathway, before and after controlling for the mediator, using the Sobel test.[78,79] Women who are lost to follow-up are likely to not complete post-intervention questionnaires; therefore, appropriate missing data methods (e.g., multiple imputation, inverse probability weighting, or likelihood-based methods) will be employed to account for unobserved mediator variables. If the intervention is not found to be effective, we will still conduct the mediation analysis to examine effect of suppression (i.e., if the intervention increases one belief but reduces another, these changes could offset one another in how they affect behavior).

AIM 3. Estimate the incremental cost per additional woman completing screening of adding at-home HPV self-collection to enhanced reminders.

Overall approach: Cost assessment is necessary for program planning to determine whether investment in at-home HPV self-collection among women receiving enhanced reminders provides meaningful clinical benefit at a reasonable cost. *We propose here to directly measure costs and a surrogate marker of prevention effectiveness (women screening complete) in the context of a specific RCT.* High internal validity of study results and the ability to collect detailed cost data and primary outcomes in the context of a well-designed RCT provide concrete advantages over simulation modeling studies, although this approach is more limited in terms of generalizability. There is substantial precedent for trial-based economic analyses to use incremental cost per additional person screened as the primary measure of cost-effectiveness.[105-110,122,133-136,140] Following previously published methods, we will measure the non-research-related costs associated with the intervention and control arms to estimate incremental cost per additional patient completing screening in each arm. Analysis will take the perspective of public payers, which absorb many of the cancer-related screening and treatment costs for uninsured and publicly insured women through Medicaid, Medicare, and government subsidization of safety net providers.

3a. Measure economic inputs required for the intervention and control arms

Measures and data collection: Cost assessment will estimate the total costs required to coordinate and administer (i) at-home HPV self-collection followed by enhanced reminder (intervention arm) and (ii) enhanced reminder alone (control arm). In both arms, we will record the amount of time in minutes spent on each enhanced reminder phone call (and attempts to complete this call), including HPV results delivery and appointment scheduling when relevant, and calculate cost by multiplying total time spent (in 60 minute blocks) with the average hourly salary of the clinic nurses, administrative staff, and ASHA agents making these calls. We will similarly assess time spent in training, sample handling, and data management. In the intervention arm, we will record costs associated with self-collection kit materials and laboratory testing. Mailing-associated costs will be recorded for both arms. We will also assess costs associated with additional health care utilization post-screening (e.g. abnormal cytology, colposcopy and treatment visits). We will not assess fixed costs common to both arms (e.g., clinic operations overhead), or costs related to the conduct of the study alone

(e.g., data collection). Indirect patient costs, such as transportation, will not be included in analysis due to difficulty standardizing and collecting such measures.

3b. Assess incremental cost of at-home HPV self-collection per additional woman screened.

Precision of estimated incremental cost per additional woman completing screening is constrained by sample size for the primary outcome. As detailed by Willan and O'Brien,[104] whether or not a given intervention is "cost-effective" is dependent on a specified *willingness-to-pay* (WTP) threshold. Power of a given study to determine whether an intervention is cost-effective using traditional statistical approaches is a function of the intervention's effect size, but also of the WTP. Unlike analyses based on commonly accepted WTP thresholds of cost per quality-adjusted life year (QALY) saved, there is no standard, commonly accepted WTP threshold for cost per additional woman screened.[127] Reported ranges of WTP for US-based programs to increase colorectal cancer screening range from less than $100 to over $5000 per additional person screened.[105-108] *For programs to increase cervical cancer screening uptake, only European estimates are available.*[109,110]

We will use a Bayesian approach to economic analysis, employing net monetary benefits[111-113] given the uncertainty about what a decision-maker would consider an appropriate WTP threshold, and the inherent uncertainty in the estimates of incremental costs and effectiveness resulting from the trial (as measured by the uncertainty surrounding the mean values from the trial for each of these parameters). The net monetary benefit (NMB) of an intervention will be defined as $\boldsymbol{Effectiveness * WTP - Costs}$, where WTP is defined as the threshold value of incremental cost per incremental unit of effectiveness that is considered "acceptable" to decision-makers. When comparing 2 or more interventions, the "optimal" choice is the one that has the highest NMB at a given value for WTP. For example, at a WTP of $0, the least expensive option will always be favored, whereas at very high values of WTP, the most effective option will be favored. Reporting NMB at different potential values of WTP allows decision-makers to determine whether to implement at-home HPV self-collection in their settings based on their own WTP per additional woman screened.

We will define effectiveness as the proportion of women completing screening in each arm. This parameter will have a range of potential values drawn from our trial results, defined by a beta distribution, with costs for each arm defined by a distribution appropriate to the shape of the data. Using Monte Carlo simulation with 10,000 iterations, we will estimate joint distributions of costs and effectiveness (i.e., women screening complete) for each arm; estimate proportion of iterations where adding HPV self-collection produces the highest NMB at varying levels of WTP; and present results using cost-effectiveness acceptability curves, where y-axis represents proportion of simulations and x-axis represents a range in decision makers' WTP.[114-116]

Future work beyond the scope of current grant application: These analyses will allow for future studies to integrate data collected in this proposed study into simulation models to estimate longer-term health and economic impacts of the intervention compared to other evidence-based cervical cancer prevention strategies.[123,124] Future work will estimate cost-effectiveness in terms of other outcomes (e.g., cancer cases and deaths prevented, quality-adjusted life expectancy, etc.) and short- and medium term budget impact (expected costs and health outcomes over a 5-10 year time horizon in a specific population) of implementing the intervention. Simulation models synthesizing data from different study types are often used to determine both budget impact[117] and cost-effectiveness.[118]

Dissemination. The research team will develop a minimum of 2-3 manuscripts per aim (minimum 6-9 total) for submission to peer-reviewed journals, including JAMA and the International Journal of Cancer. Results will also be submitted for presentation at international meetings, including the International Papillomavirus Conference, Eurogin, the Society for Medical Decision Making, and the American Public Health Association.

**TIMELINE**	**Year 1**				**Years 2-3**	**Year 4**				**Year 5**			
Study component	Q1	Q2	Q3	Q4		Q1	Q2	Q3	Q4	Q1	Q2	Q3	Q4
IRB approval (pre–study implementation)													
Hiring of study staff	■												
Development of recruitment materials	■	■											
Hiring and training of outreach coordinators	■	■											
Training and preparation of clinics		■	■										
Participant recruitment, pre-intervention questionnaire, and enrollment at ~24 women per month			■	■	■	■	■						
Implementation of RCT			■	■	■	■	■	■	■				
Clinic sample collection and laboratory testing					■	■	■	■	■				
Follow-up surveys					■	■	■	■	■				
Clinic visit tracking					■	■	■	■	■				

TIMELINE	Year 1				Years 2-3	Year 4				Year 5			
Study component	Q1	Q2	Q3	Q4		Q1	Q2	Q3	Q4	Q1	Q2	Q3	Q4
Manuscript preparation and results dissemination													

Alternative approaches and limitations.

Definition of study arms. A 3-arm study would have allowed comparison with HPV self-collection alone, or with a non-active (standard of care) control. We selected a 2-arm design to balance maximum statistical power for assessing effect with sample size that was realistic to recruit during the study period. Rather than comparing to current standard of care, we wanted to see if HPV self-collection added any relative benefit beyond an existing evidence-based intervention (enhanced reminders) that is already used by many private clinics and many BCCCP programs for women who are due or overdue. Our proposed study is designed to determine if HPV self-collection adds sufficient benefit to justify the added expense and effort.

Behavioral constructs. We considered sending control arm participants a mailing at the same time as the kit is mailed to the intervention arm, an approach that is commonly used to control for a possible effect of the receipt of a mailing itself. We chose not to take this approach because we want to compare the interventions as they could be implemented in the real world. We acknowledge that the phone call to complete the post-intervention questionnaire after the enhanced reminder call might act as an additional screening reminder potentially creating higher overall in-clinic screening than in real practice. However, this effect should be equal between the two arms, and it is important to the validity of the mediation analysis to measure psychological mediating variables *before* in-clinic screening is completed, to maintain temporality of predictors and outcomes: otherwise we cannot know whether changes in mediators precede or follow completion of in-clinic screening.

HPV vaccination. Prophylactic vaccination is expected to reduce future population rates of high-grade cervical disease and cancer by reducing incidence and persistence of oncogenic HPV types 16 and 18.[119] However, screening remains critical to cervical cancer prevention, as many women will remain unvaccinated and current generation prophylactic vaccines do not cover all oncogenic HPV types causing cancer.

Outcome measures. *We chose to define our primary outcome, screening completion, as in-clinic screening or receipt of a negative HPV self-collection result, thus considering women with HPV negative results by self-collection to be screening complete. Self-collection has comparable sensitivity to physician collection for the detection of high-grade CIN-2+, is more sensitive than cytology alone for CIN2+ detection*, and should not require confirmation by physician HPV results.[8,31] *High-risk HPV self-collection could be used alone for primary screening, yet follow-up with Pap smear is required to obtain higher specificity for CIN-2+ detection.* Self-collection for HPV testing is not yet FDA-approved for US clinical use. *Our partner clinics provide both cytology alone and co-testing for women 30 years and older per USPSTF recommendations and either of these screening options is sufficient for our outcome of in-clinic screening.*[3]

Self-collection FDA approval: Our primary analysis estimates the effect of the intervention as it would be implemented if HPV self-collection were approved for primary screening. Although HPV self-collection is not yet FDA approved as a primary screening test, physician-collected HPV testing was recently approved by the FDA for primary screening for US women 30 years and older, with triage of high-risk HPV positives to cytology. Diagnostic manufacturers are now considering HPV self-collection clinical trials to seek future FDA approval.

Assessing mediation of in-clinic screening completion. *Because self-collection HPV negative women are considered screening complete, we cannot examine perceived risk as predictor of in-clinic screening uptake in all trial participants.* However, our highest priority is to evaluate self-collection as it would be implemented in practice, with HPV negative women considered screening complete. In-clinic screening is most needed for women with HPV positive or unknown HPV status, and our analyses address these groups.

Barriers among population of interest. Though self-collection does not remove all barriers to screening, it still has the potential for meaningful impact on our high-risk target population. *Self-collection negative participants (estimated 85% of women who return self-collected samples) will not need to complete in-clinic screening. For these women, self-collection will have removed barriers associated with in-clinic screening (e.g., embarrassment, transportation, and time). Although HPV positive women will still face barriers to in-clinic screening, we expect that the increase in perceived risk of cervical cancer from learning their HPV positive status will increase their motivation to overcome these barriers.* Our study population of low-income, uninsured and publically insured women is eligible for free cervical cancer screening under BCCCP or Medicaid. Because the receipt of study incentives is not linked to clinic attendance, there are no financial incentives to screening completion. Therefore, the study will provide insight into whether HPV self-collection generates sufficient motivation for these women to overcome non-economic barriers to attend in-clinic screening.
