# Supplementary material for: Streamlined Self-Collection Screening for Sexually Transmitted Infections and Human Papillomavirus: A Single-Group Secondary Analysis of a Randomized Clinical Trial
Source: JAMA Netw Open. 2026 Jan 8;9(1):e2551345. doi: 10.1001/jamanetworkopen.2025.51345 (PMC12784235; doi:10.1001/jamanetworkopen.2025.51345)
Supplement: Supplement 2. — eFigure 1. Study Flow Diagram eFigure 2. Sexually Transmitted Infections Among Participants With Positive Tests From Mailed Self-Collection Kits eMethods. Participant Mailed Information and Instructions [file jamanetwopen-e2551345-s002.pdf]

Supplemental Online Content

Ganguly AP, Pretsch PK, Brewer NT, et al. Streamlined self-collection screening for sexually transmitted infections and human papillomavirus: a secondary analysis of a randomized clinical trial. *JAMA Netw Open.* 2026;9(1):e2551345.
doi:10.1001/jamanetworkopen.2025.51345

eFigure 1. Study Flow Diagram

eFigure 2. Sexually Transmitted Infections Among Participants With Positive Tests From Mailed Self-Collection Kits

eMethods. Participant Mailed Information and Instructions

This supplemental material has been provided by the authors to give readers additional information about their work.

© 2025 Ganguly AP et al. *JAMA Network Open*.

eFigure 1. Study Flow Diagram

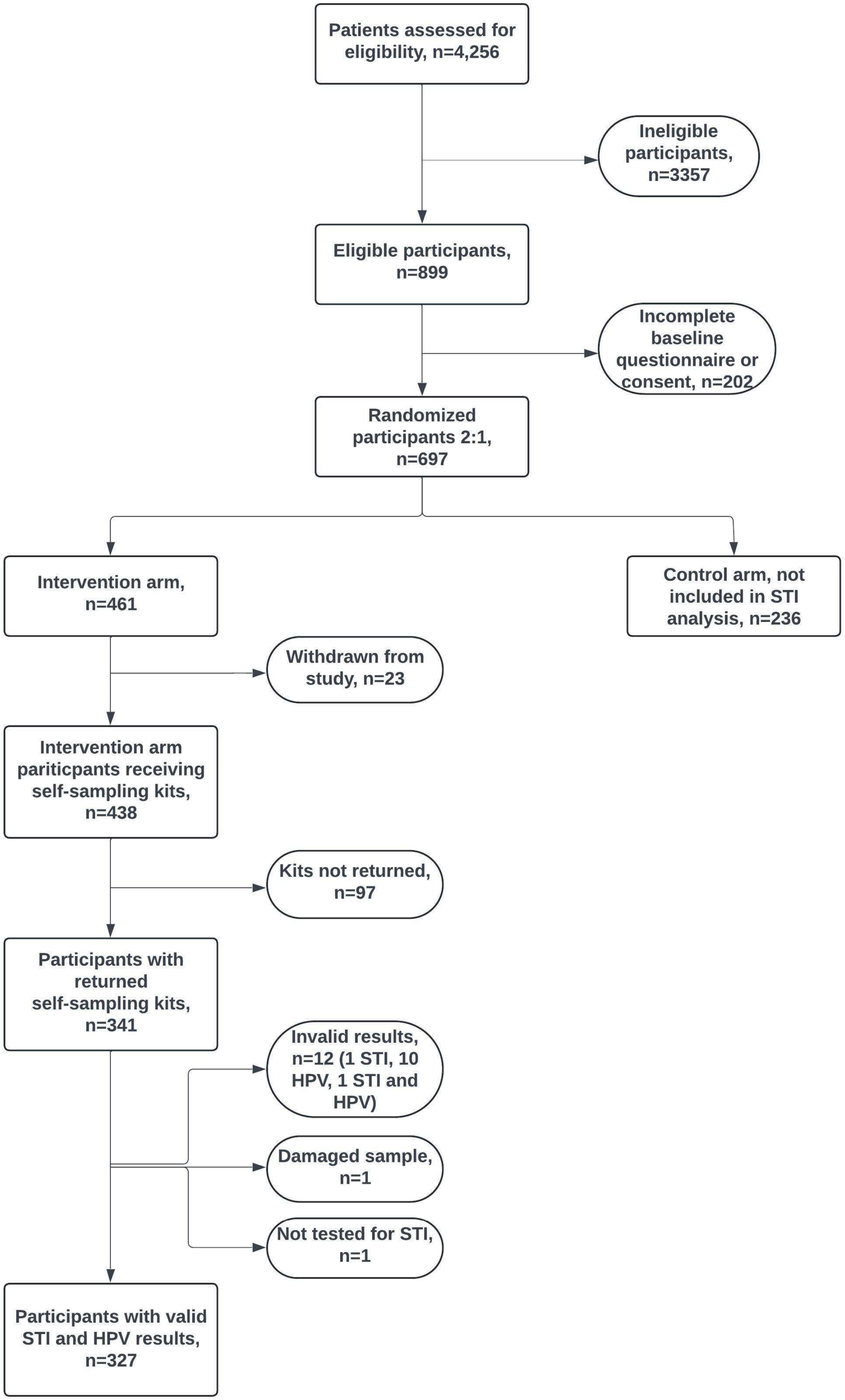

© 2025 Ganguly AP et al. *JAMA Network Open*.

eFigure 2. Sexually Transmitted Infections Among Participants With Positive Tests From Mailed Self-Collection Kits

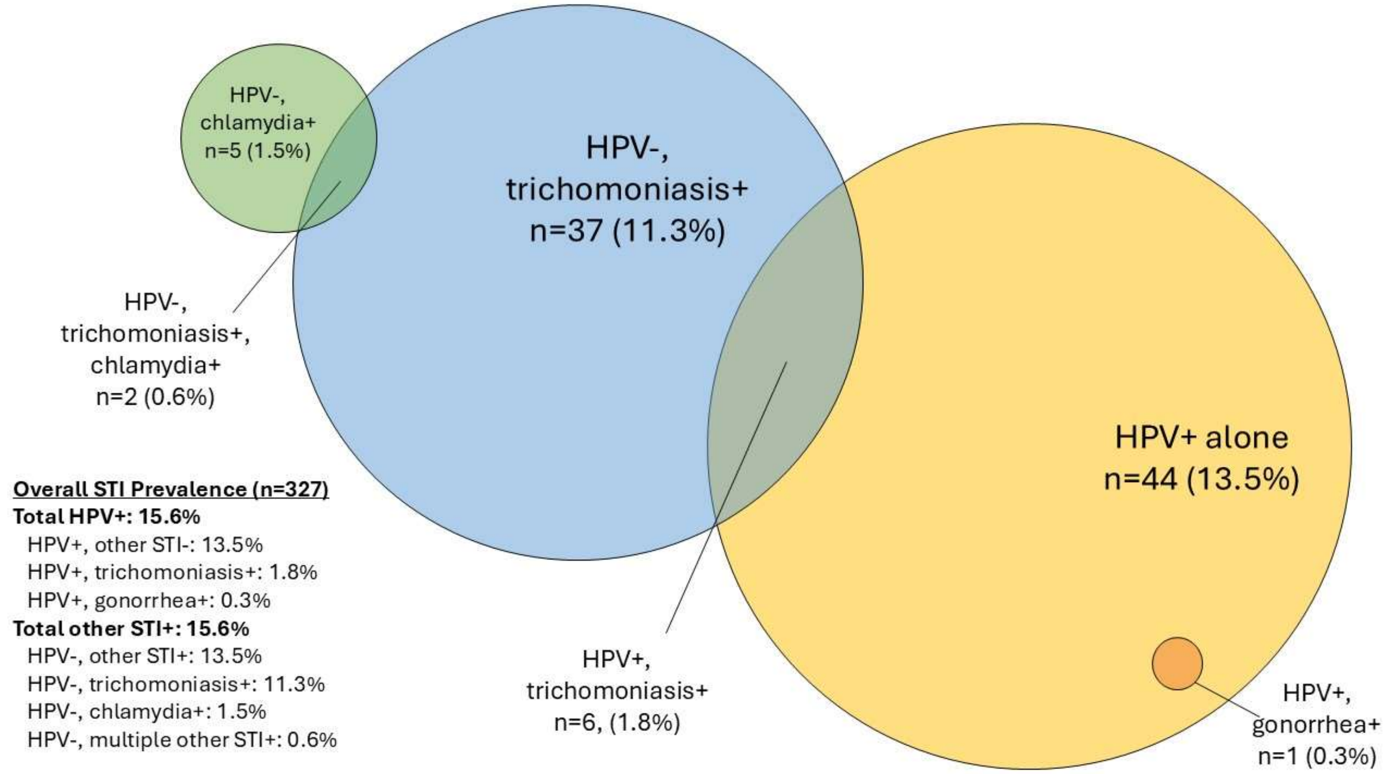

© 2025 Ganguly AP et al. *JAMA Network Open*.

My Body! My Test!

Things to Know about the Self-Test

Learn about your risk for cervical cancer.

This test looks for human papillomavirus (HPV), the virus that can cause cervical cancer. Many doctors already test women for HPV in the clinic. This self-test is a simple and convenient way to test for HPV at home.

Screening can prevent cervical cancer.

Testing for HPV is a way to test for cervical cancer. A Pap smear is another way to test for cervical cancer. If screening finds a problem early, the problem can almost always be treated before it becomes serious.

The self-test is safe and accurate.

Studies show that HPV self-testing is very good at telling if a woman is at higher risk of getting cervical cancer. Women all over the world have used the self-test. It is safe for women to use at home.

You have a choice.

We encourage you to get screened for cervical cancer to protect your health. Using the self-test in the privacy of your own home is a great way to do this. But, you can go to the clinic for a Pap smear instead if you want to: Call us to schedule a free appointment.

Understanding your HPV self-test results

If you **have** an HPV infection, you are at higher risk for getting cervical cancer. You should go to a clinic right away to get a Pap smear.

If you **don't have** an HPV infection, it is probably safe to wait 2 years before getting another HPV test or Pap smear.

Questions?

(919) 966-6766

mybodymytest@unc.edu

More details on back

© 2025 Ganguly AP et al. *JAMA Network Open*.

How is the self-test different from a Pap smear?

The self-test looks for HPV, the virus that can cause cervical cancer. In comparison, a Pap smear done by a doctor looks for abnormal (unhealthy) cells on the cervix that could be cancer, or could turn into cancer. Depending on your results, you may need follow-up care from a doctor.

What if my self-test says I have HPV?

HPV is very common. Most women who have HPV stay healthy. But, having HPV puts a woman at higher risk for cervical cancer. **If you have HPV, you should get a Pap smear as soon as possible**. We will help you make a free appointment at a local clinic. Your Pap smear might find that everything is ok. If the Pap smear finds a problem, we will help you get the care you need even if you can't afford to pay for it yourself.

What if my self-test says I don't have HPV?

If your self-test is negative (the test doesn't find HPV), it is probably safe to wait 2 years before getting screened again. Studies show that the self-test is very accurate, but no test is perfect. If you want to double-check your self-test result, we will help you schedule a free Pap smear at a local clinic.

How often should I get screened for cervical cancer?

All women should get regular screening to prevent cancer, no matter what their HPV result is. Women 21 to 29 years old should get a Pap smear every 3 years. Women 30 or older should get a Pap smear and HPV test together every 5 years (or a Pap smear every 3 years). Getting a Pap smear and HPV test at the same time is the best way to screen. If you are 30 or older, ask your doctor to do this the next time you get screened. Women with HIV, or who have had an abnormal Pap smear might need to get screened more often.

What else will the self-test tell me?

If you gave us permission when you signed the consent form, we will also test your sample for 3 common sexually transmitted diseases (STDs): gonorrhea, chlamydia, and trichomonas vaginalis. If you have any of these infections, we will help you get free treatment. These infections are easy to treat.

How can I learn more?

Visit our website: www.mybodymytest.org
Call our project coordinator, Sarah Doughty: (919) 966-6766, or
email her at mybodymytest@unc.edu

© 2025 Ganguly AP et al. *JAMA Network Open*.

My Body! My Test!

Questions? Comments?
(919) 966-6766
www.mybodymytest.com
mybodymytest@unc.edu

Self-Test Instructions

Do not collect a sample if you are pregnant or on your period.

If you think you might be pregnant, please call us.
If you are on your period, please wait until you have stopped bleeding for at least one day.

Before you start…

Make sure you have in your kit:

- a brush with a blue handle
- a small tube with liquid in it.

You will also need an empty cup to put the tube in, so that it does not fall over.

Next, follow these steps…

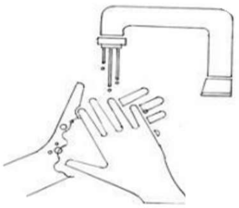

1. Wash your hands well.

2. Open the tube by twisting off the lid.
   Do not pour out the liquid. Save the lid. Set the tube in the cup so it doesn't fall over.

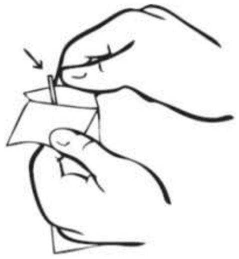

3. Open the envelope with the brush in it.
   Remove the brush.
   Try not to touch the white brush tip with your hands.

Continue on back

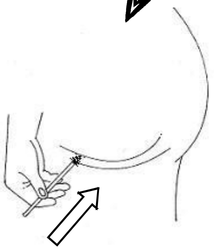

© 2025 Ganguly AP et al. *JAMA Network Open*.

4. Stand, sit, or lie down in a comfortable position.
   Some women find it helpful to squat with their
   legs apart.

5. Relax and gently push the brush into your vagina until you feel resistance. It is kind of like putting in a tampon.

6. Turn the brush around 5 times while it is inside your vagina.

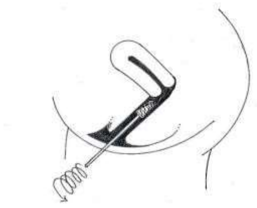

7. Slowly pull out the brush.
   Try not to touch the white brush tip with your fingers.

8. Notice that there is clear plastic on the handle near the white brush tip.
   Push the clear plastic down so that the white brush tip falls into the tube.

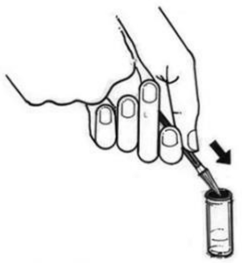

9. Put the lid back on the tube.
   Screw the lid on <u>tightly</u>.

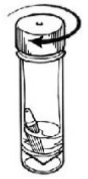

10. Put the tube in the clear plastic bag and close the bag.

11. Write the time and date that you collected the sample on the Self-Test Follow-Up Form

You are all done!

© 2025 Ganguly AP et al. *JAMA Network Open*.
