# Supplementary material for: Streamlined Self-Collection Screening for Sexually Transmitted Infections and Human Papillomavirus: A Single-Group Secondary Analysis of a Randomized Clinical Trial
Source: JAMA Netw Open. 2026 Jan 8;9(1):e2551345. doi: 10.1001/jamanetworkopen.2025.51345 (PMC12784235; doi:10.1001/jamanetworkopen.2025.51345)
Supplement: Supplement 3. — Data Sharing Statement [file jamanetwopen-e2551345-s003.pdf]

Data Sharing Statement

Ganguly. Streamlined Self-Collection Screening for Sexually Transmitted Infections and Human Papillomavirus. *JAMA Netw Open*. Published January 08, 2026.
doi:10.1001/jamanetworkopen.2025.51345

Data
Additional Information: ClinicalTrials.gov Identifier NCT02651883.
Data available: Yes
Data types: Deidentified participant data
How to access data: Inquiries regarding data request should be made to Dr. Smith at jsssmith@email.unc.edu.
When available: With publication

Supporting Documents
Document types: None

Additional Information
Who can access the data: Investigators interested in accessing these data for the purposes of future studies can do so under the following conditions: Institutional Review Board approval has been obtained from the University of North Carolina at Chapel Hill, Chapel Hill, NC, USA, the institution covering the investigator; data security procedures ensuring patient privacy have been shown by the investigator; and a Data Use Agreement has been filled. Final datasets for analysis will not include any identifying information.
Types of analyses: Secondary analyses approved by the study team.
Mechanisms of data availability: Investigators interested in accessing these data for the purposes of future studies can do so under the following conditions: Institutional Review Board approval has been obtained from the University of North Carolina at Chapel Hill, Chapel Hill, NC, USA, the institution covering the investigator; data security procedures ensuring patient privacy have been shown by the investigator; and a Data Use Agreement has been filled. Final datasets for analysis will not include any identifying information.
